# Experimental Investigation of Laser-Assisted Ultrasonic Vibration Milling of CF/PEEK-Based CFRTP Unidirectional Laminates

**DOI:** 10.3390/mi17091087

**Published:** 2026-09-16

**Authors:** Zhengqiang Li, Kang Xiao, Minghai Wang, Qijia Wang, Xuezhi Wang, Xianjun Kong, Siyu Zhou

**Affiliations:** 1 College of Mechanical and Electrical Engineering, Shenyang Aerospace University, Shenyang 110136, China; xk13841273893@163.com (K.X.); yuyigraduatessau01@163.com (M.W.); 18602435337@163.com (Q.W.); wangxuezhineu@126.com (X.W.); 18143695773@163.com (X.K.); zhousiyu@sau.edu.cn (S.Z.); 2 Key Laboratory of Rapid Development & Manufacturing Technology for Aircraft, Shenyang Aerospace University, Ministry of Education, Shenyang 110136, China

**Keywords:** carbon fiber-reinforced polyetheretherketone, laser–ultrasonic vibration-assisted milling, cutting force, surface quality

## Abstract

Carbon fiber-reinforced polyetheretherketone is widely used in aerospace, transportation, and medical devices because of its high interlaminar and intralaminar fracture toughness, excellent mechanical properties, and long-term stability at elevated temperatures. However, its two-phase structure, consisting of brittle fibers embedded in a tough matrix, presents significant machining challenges. In this study, a two-stage laser–ultrasonic vibration-assisted milling process was investigated by combining laser-induced matrix thermal softening and decomposition with high-frequency, intermittent tool–workpiece contact. Unidirectional laminates with 0° and 90° fiber orientations were machined to systematically evaluate the effects of processing parameters on cutting force, measured surface temperature, surface roughness, and machining defects. The proposed process reduced the cutting force by 7.4–26.1%, improved surface roughness, and reduced observable machined surface defects. Appropriate laser and ultrasonic parameters were associated with lower measured surface temperatures and fewer observable fiber fracture and fiber–matrix separation features, thereby improving machined surface quality. These findings clarify the synergistic effects of laser and ultrasonic parameters and provide a basis for process optimization and high-quality machining of carbon fiber-reinforced polyetheretherketone composites.

## 1. Introduction

Carbon fiber-reinforced thermoplastic composites (CFRTP) are increasingly used in aerospace, transportation and biomedical engineering owing to their high specific strength, fracture toughness, damage tolerance, repairability and recyclability [1,2]. Among these materials, carbon fiber-reinforced polyetheretherketone (CF/PEEK) is distinguished by the mechanical performance, thermal stability and chemical resistance of the PEEK matrix [1,3]. Despite near-net-shape forming, secondary machining remains necessary to remove excess material and meet dimensional accuracy, surface quality and assembly requirements [1,2,3].

The heterogeneous and anisotropic fiber–matrix structure of CFRTP gives rise to machining behaviour distinct from that of homogeneous materials. Compared with thermoset carbon fiber-reinforced polymers, the greater ductility and temperature sensitivity of thermoplastic matrices alter fiber support, chip formation, cutting force, and surface–subsurface damage [1,4,5]. Temperature-controlled orthogonal cutting by Ge et al. [4] showed that temperature-dependent changes in the matrix and interface can alter material removal and damage evolution in CFRTP. Wang et al. [5] further developed an elasto-plastic cutting model for CF/epoxy and CF/PEEK, highlighting the role of matrix plasticity in predicting cutting force and subsurface damage. Consequently, CF/PEEK machining is prone to fiber pull-out, interfacial debonding, burrs, matrix smearing, cavities and thermally induced surface damage, compromising dimensional accuracy and structural integrity [1,5].

Conventional machining of CF/PEEK has been improved through parameter optimisation, thermal control, predictive modelling and tool design. Cao et al. [6] applied a GA-BP neural network to optimise machining parameters for high-speed dry milling of UD-CF/PEEK. Song et al. [7] predicted surface roughness by incorporating machining kinematics, dynamics and carbon-fiber distribution into the model. Liu et al. [8] incorporated cutting temperature as a constraint in process optimisation to improve machining efficiency while reducing surface defects. Liu et al. [9] formulated an analytical thermal model incorporating anisotropy and temperature-dependent thermal conductivity to predict the spatiotemporal temperature distribution during high-speed dry milling of UD-CF/PEEK. Ju et al. [10] designed a milling cutter to enhance shear cutting, thereby reducing surface-layer damage and roughness. Collectively, these studies highlight the importance of controlling mechanical and thermal effects to improve the machining quality of CF/PEEK.

Beyond conventional process optimisation, assisted machining has been explored to tailor cutting conditions and material removal in fiber-reinforced composites. Laser assistance can locally modify material properties before or during cutting, thereby reducing cutting resistance under suitable conditions. Kong et al. [11] showed that laser-assisted cutting modifies fiber fracture and reduces cutting force in CFRP, with corresponding changes in material removal and surface damage. For CF/PEEK, Wang et al. [12] examined the effects of fiber orientation, laser power and spindle speed during laser-assisted milling, identifying parameter combinations that reduced cutting force and improved surface quality. A subsequent numerical and experimental study [13] clarified fiber-removal mechanisms and surface damage during laser-assisted milling of CF/PEEK. Collectively, these studies confirm the potential of laser assistance but also reveal its sensitivity to processing parameters and fiber orientation. Ultrasonic vibration-assisted machining (UAM) offers an alternative route to improve CF/PEEK machinability by altering tool–workpiece interaction and fiber fracture. Zhang et al. [14] established a fiber fracture mechanism and milling-force model for CF/PEEK, revealing the effects of high-frequency vibration on fiber deformation and fracture. Wang et al. [15] showed that longitudinal–torsional ultrasonic vibration reduced cutting force, surface roughness, and machining damage during CF/PEEK milling. Zhang et al. [16] developed a large-amplitude longitudinal ultrasonic milling system and evaluated its effects on cutting force, temperature and surface quality of UD-CF/PEEK. Collectively, these studies demonstrate the benefits of ultrasonic assistance for CF/PEEK machining, although its effectiveness depends on vibration characteristics and fiber orientation.

Laser and ultrasonic assistance have been combined to modify the thermal–mechanical conditions during composite machining. Previous studies have demonstrated the feasibility and potential advantages of this hybrid-assisted strategy. Studies on difficult-to-machine particle-reinforced metal-matrix composites showed that laser–ultrasonic assistance can reduce cutting force and improve surface integrity [17,18]. This hybrid approach has also been extended to CF/PEEK. Xiao et al. [19] compared conventional, laser-assisted, ultrasonic-assisted, and laser–ultrasonic-assisted milling of CF/PEEK under consistent cutting conditions. Laser–ultrasonic assistance reduced cutting force and machining damage relative to conventional and single-assisted milling. Wang et al. [20] examined the effects of machining strategy and fiber orientation on cutting force, energy, temperature, surface roughness, and damage.

Although laser–ultrasonic-assisted machining of CF/PEEK has demonstrated promising performance, previous studies have mainly focused on comparisons among machining strategies, fiber orientations, spindle speeds, and their effects on material removal and surface damage [19,20]. However, the individual effects of key laser and ultrasonic parameters remain insufficiently quantified. In particular, the effects of laser power, scanning speed, vibration frequency and amplitude on cutting force, temperature, surface roughness and machining-induced damage have not yet been systematically quantified under consistent milling conditions. Establishing these parameter–response relationships is important for understanding machining responses and guiding process-parameter selection.

Building on these previous comparative studies, the present work investigates a two-stage laser–ultrasonic vibration-assisted milling process for unidirectional CF/PEEK laminates with 0° and 90° fiber orientations. The individual effects of laser power, scanning speed, vibration frequency, and amplitude are evaluated using a one-factor-at-a-time design under fixed mechanical milling conditions. Cutting force, infrared-measured apparent surface temperature, areal surface roughness, and machining-induced surface damage are evaluated to determine the corresponding parameter–response relationships. The resulting trends provide an experimental basis for selecting laser and ultrasonic parameters to improve the machined surface quality of CF/PEEK.

## 2. Experimental Materials and Methods

### 2.1. Material Removal Process Under Pulsed Laser Irradiation

**Figure 1:**
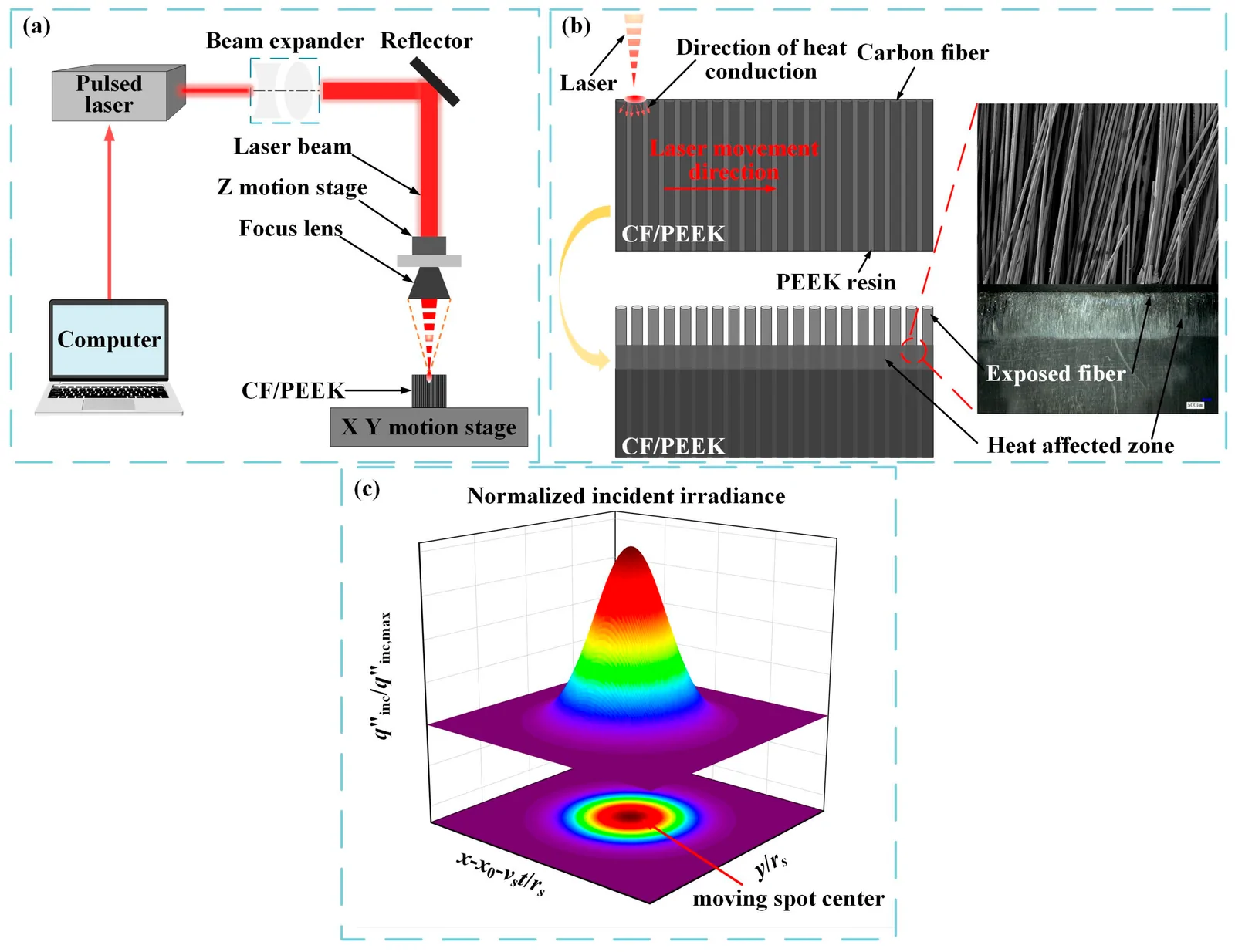
Schematic illustration of the laser irradiation process: (**a**) laser-scanning configuration; (**b**) schematic representation of thermal modification of CF/PEEK during laser irradiation; and (**c**) normalized spatial intensity distribution of the assumed Gaussian laser source.

Figure 1a illustrates the pulsed-laser irradiation process. During irradiation, the workpiece moved along the scanning direction, with the laser beam normal to the CF/PEEK surface. As shown in Figure 1b, localized laser heating thermally modified the predefined region before ultrasonic vibration-assisted milling. The surface irradiance was approximated by a circular Gaussian distribution, a common representation of laser-beam intensity [21,22]. Because the beam profile was not measured using a beam profiler, the Gaussian distribution was treated as a modelling assumption. The surface spot radius was estimated using a graduated ruler as *r_s_* ≈ 1.5 mm. For a laser beam scanning along the x-direction at a constant speed *v_s_*, the incident irradiance is expressed as

(1) $$q_{\mathrm{inc}}^{\prime \prime }(x,y,t)=\frac{2P(t)}{\pi r_{s}^{2}}e^{\left[-\frac{2({\left(x-x_{0}-v_{s}t\right)}^{2}+y^{2})}{r_{s}^{2}}\right]}$$

where $q_{\mathrm{inc}}^{\prime \prime }$ is the incident irradiance (W/mm^2^); *P*(*t*) is the instantaneous laser power (W); *x*, *y*, *x*_0_, and *r_s_* are expressed in mm; *v_s_* is the scanning speed (mm/s); and *t* is time (s). Equation (1) is based on the standard Gaussian beam-intensity distribution, with the term *x*-*x*_0_-*v_s_t* accounting for the motion of the laser spot.

For a periodic pulsed laser, the temporal variation is contained in *P*(*t*). The pulse period *T*, repetition frequency *f_p_*, pulse duration *τ_p_*, and duty cycle *D* satisfy

(2) $$T=\frac{1}{f_{p}},\;D=f_{p}\tau_{p},\;E_{p}=\frac{P_{\mathrm{avg}}}{f_{p}}$$

where *f_p_* is expressed in Hz, *T* and *τ_p_* in s, *D* is dimensionless, *P*_avg_ is the average laser power (W), and *E_p_* is the energy per pulse (J) [23]. For an ideal rectangular pulse, *E_p_* = *P*_pk_*τ_p_*, where *P*_pk_ bis the peak laser power (W), and therefore *P*_avg_ = *DP*_pk_. These latter relationships are used only as ideal pulse definitions and do not imply that the actual temporal pulse shape was rectangular.

The repetition frequency also determines the spacing between consecutive pulses during scanning. The pulse-to-pulse spacing and nominal geometrical overlap are given by:

(3) $$\Delta x=\frac{v_{s}}{f_{p}},\;n_{p}=1-\frac{\Delta x}{2r_{s}}=1-\frac{v_{s}}{2r_{s}f_{p}}$$

for Δ*x* < 2*r_s_*, where Δ*x* is the centre-to-centre pulse spacing (mm) and *η_p_* is the dimensionless pulse overlap ratio [24]. When Δ*x* > 2*r_s_*, the nominal geometrical overlap is zero.

In the present experiments, the pulse repetition frequency, pulse duration, and duty cycle were maintained at the preset controller settings but were not independently recorded as experimental variables. Their exact experimental values therefore cannot be reliably reconstructed retrospectively. Consequently, Equations (2) and (3) are provided to clarify how the temporal pulse characteristics enter the laser-energy description; the actual pulse energy and pulse-to-pulse overlap were not evaluated numerically. The nominal laser wavelength was 1080 ± 5 nm. Because the absorptivity of the CF/PEEK laminate at this wavelength was not independently measured, the absolute absorbed heat flux, $q_{abs}^{\prime \prime }=Aq_{inc}^{\prime \prime }$, and the total absorbed laser energy were not calculated. Accordingly, Figure 1c presents only the normalized spatial Gaussian distribution rather than an absolute absorbed heat-flux distribution.

The present formulation describes only the spatial and temporal characteristics of the incident laser source at the workpiece surface. No transient heat conduction equation was solved, and therefore thermal boundary conditions such as convection, radiation, or prescribed-temperature boundaries were not applied. The formulation is used to describe the laser irradiation conditions rather than to predict the absolute absorbed energy or three-dimensional transient temperature field.

### 2.2. Kinematics of Ultrasonic Vibration-Assisted Milling

**Figure 2:**
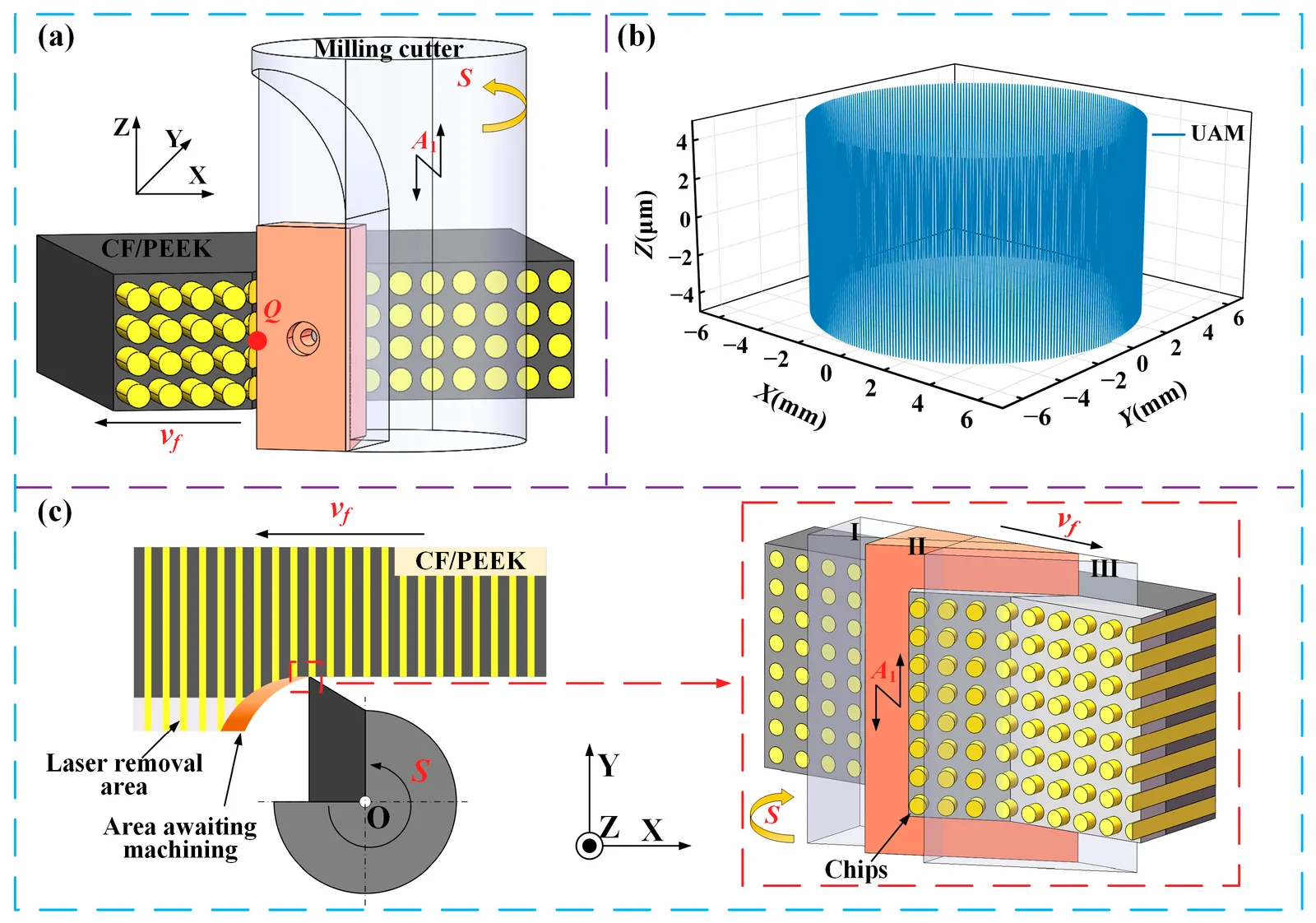
Schematic illustration and kinematic description of laser-pretreated longitudinal ultrasonic vibration-assisted milling of CF/PEEK. (**a**) principle of longitudinal UAM and coordinate system; (**b**) calculated cutting-edge trajectory under UAM; (**c**) laser pretreatment and subsequent milling process.

Figure 2a illustrates the principle of longitudinal ultrasonic vibration-assisted milling (UAM). A single-edge cutter was considered to simplify the cutting-edge trajectory analysis. A workpiece-fixed Cartesian coordinate system *0-XYZ* was defined. The +*X* axis denotes the cutter feed direction relative to the workpiece, the *Y* axis lies transverse to the feed direction within the workpiece surface, and the *Z* axis coincides with both the cutter axis and the longitudinal vibration direction. The origin *0* defines the reference position of the cutter centre at *t* = 0. For a cutter with z cutting edges, the instantaneous angular position of the *i*th edge is given by

(4) $$\theta_{i}(t)=\theta_{0}+\frac{2\pi S}{60}t-\frac{2\pi (i-1)}{z}$$

where *S* is the spindle speed (r/min), *i* is the cutting-edge index, z is the number of cutting edges, and *θ*_0_ is the initial angular position of the reference cutting edge measured from the +*X* axis. In the present trajectory calculation, *θ*_0_ = 0 was used. Since a single-edge cutter was employed experimentally, *z* = 1 and *i* = 1, the cutting-edge phase-offset term is therefore zero. The trajectory of a reference point *Q* on the cutting edge can then be expressed as

(5) $$\left\{\begin{array}{l} X_{i}(t)=X_{0}+\frac{vf}{60}t+R\cos\theta_{i}(t)\\ Y_{i}(t)=Y_{0}+R\sin\theta_{i}(t)\\ Z_{i}(t)=Z_{0}+\frac{A}{1000}\sin(2\pi ft+\psi_{0}) \end{array}\right.$$

where *R* is the cutter radius (mm), *v_f_* is the feed speed (mm/min), *A* is the longitudinal vibration amplitude (μm), *f* is the ultrasonic vibration frequency (Hz), and *t* is time (s). *X*_0_, *Y*_0_, and *Z*_0_ denote the initial coordinates of point *Q*, and ψ_0_ is the initial phase of the axial ultrasonic vibration. ψ_0_ = 0 was adopted for the calculated trajectory. The factors 1/60 and 1/1000 convert the experimentally reported feed speed from mm/min to mm/s and the vibration amplitude from μm to mm, respectively.

In Equation (5), the term *v_f_*/60 describes the translational feed motion of the cutter centre along the *X* direction; the cosine and sine terms represent the rotational motion of the cutting edge in the *XY* plane; and the sinusoidal term in the *Z* coordinate describes the longitudinal ultrasonic vibration of the cutter. The combination of these three motions produces the three-dimensional cutting edge trajectory shown in Figure 2b, with the following parameters: *R* = 6 mm, *S* = 6000 r/min, *v_f_* = 480 mm/min, *f* = 34 kHz, and *A* = 5 μm. During milling, cutter rotation causes the cutting edge to periodically engage and disengage from the workpiece, while longitudinal ultrasonic vibration alters the instantaneous tool–workpiece contact. Figure 2c illustrates the resulting material-removal process within the laser-pretreated CF/PEEK region.

### 2.3. Experimental Materials and Cutting Tools

**Figure 3:**
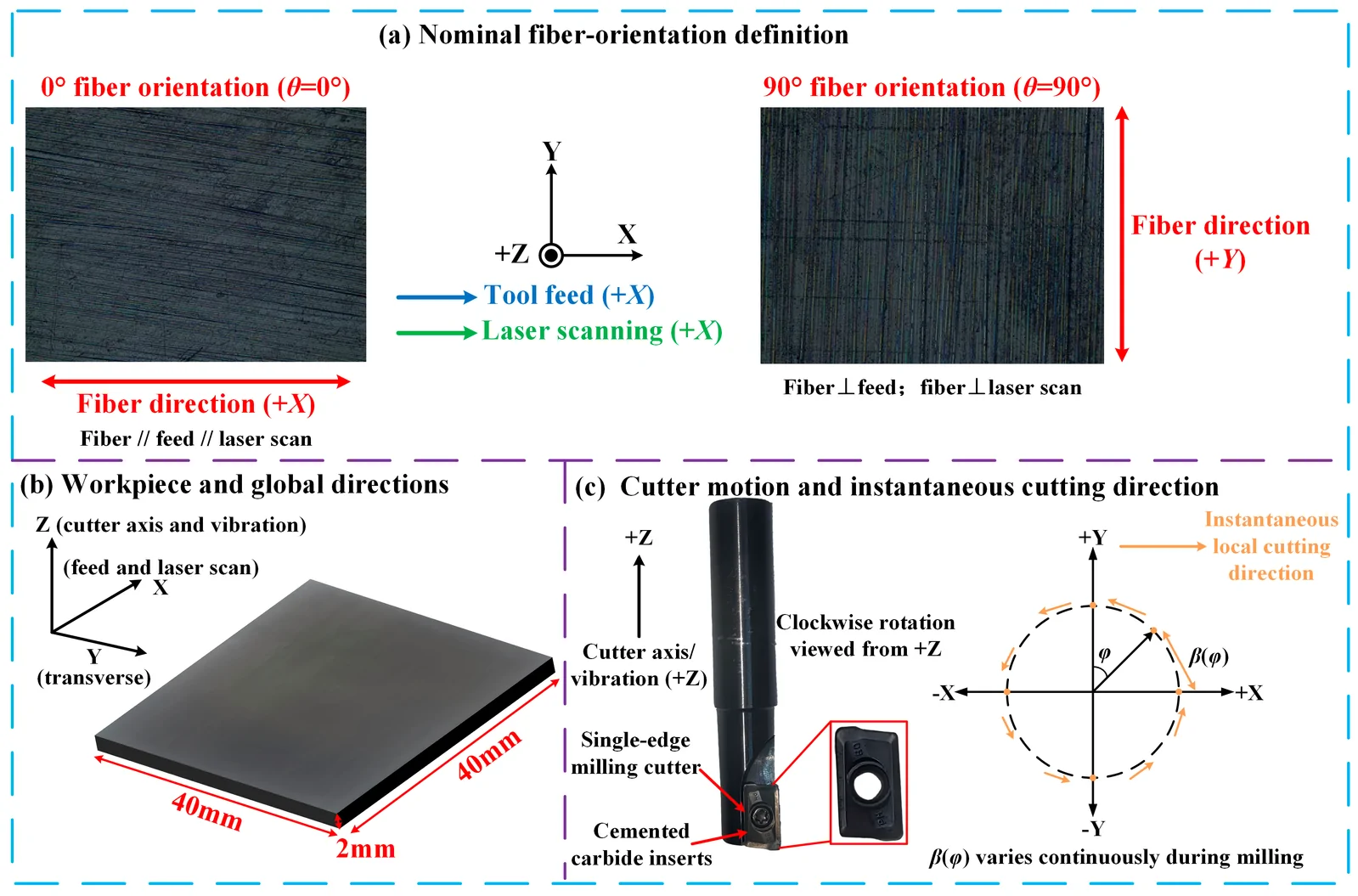
Specimens and cutting tools. (**a**) schematic of the fiber orientation angle; (**b**) CF/PEEK unidirectional laminate; (**c**) milling cutter arbor and insert.

The experiments were conducted using T700-grade CF/PEEK composites manufactured by Danyang, China; Jiangsu Hengbo Composite Materials Co., Ltd., with a fiber volume fraction of 50 ± 3 vol%. The glass transition temperature (*T*_g_) and melting temperature of the PEEK resin are 143 °C and 345 °C, respectively [25]. The detailed mechanical properties are listed in Table 1, as provided by the manufacturer. As shown in Figure 3a, the nominal fiber-orientation angle *θ* is defined in the laminate plane as the angle between the fiber axis and the macroscopic tool-feed direction (+X). The laser-scanning direction is parallel to the tool-feed direction and is also aligned with +X. Accordingly, *θ* = 0° indicates that the fiber axis is parallel to both the tool-feed and laser-scanning directions, whereas *θ* = 90° indicates that the fiber axis is aligned with +Y and perpendicular to these directions. The XY plane corresponds to the machined top surface of the workpiece, while the Z direction is normal to this surface and coincides with the cutter axis and the longitudinal ultrasonic-vibration direction. It should be emphasized that *θ* denotes the nominal global fiber orientation. During milling, the instantaneous local cutting direction is tangential to the cutter trajectory and changes continuously with the cutter angular position φ. Consequently, the instantaneous angle *β*(*φ*) between the fiber direction and the local cutting direction also varies continuously, even when the nominal fiber orientation is fixed at 0° or 90°. Unidirectional plates with dimensions of 300 mm × 300 mm × 2 mm were fabricated by hot compression moulding and subsequently cut into workpieces of 40 mm × 40 mm × 2 mm using a diamond wire saw, as shown in Figure 3b. In the experiments, a 12 mm-diameter single-tooth square-shoulder end mill fitted with an APKT11T308-APM cemented-carbide insert (YBS203 grade, ZCC-CT, Zhuzhou, China), featuring a positive cutting geometry, a nominal clearance angle of 11°, and a corner radius of 0.8 mm, was used, as shown in Figure 3c.

### 2.4. Experimental Design and Experimental Procedure

#### 2.4.1. Experimental Setup and Procedure

**Figure 4:**
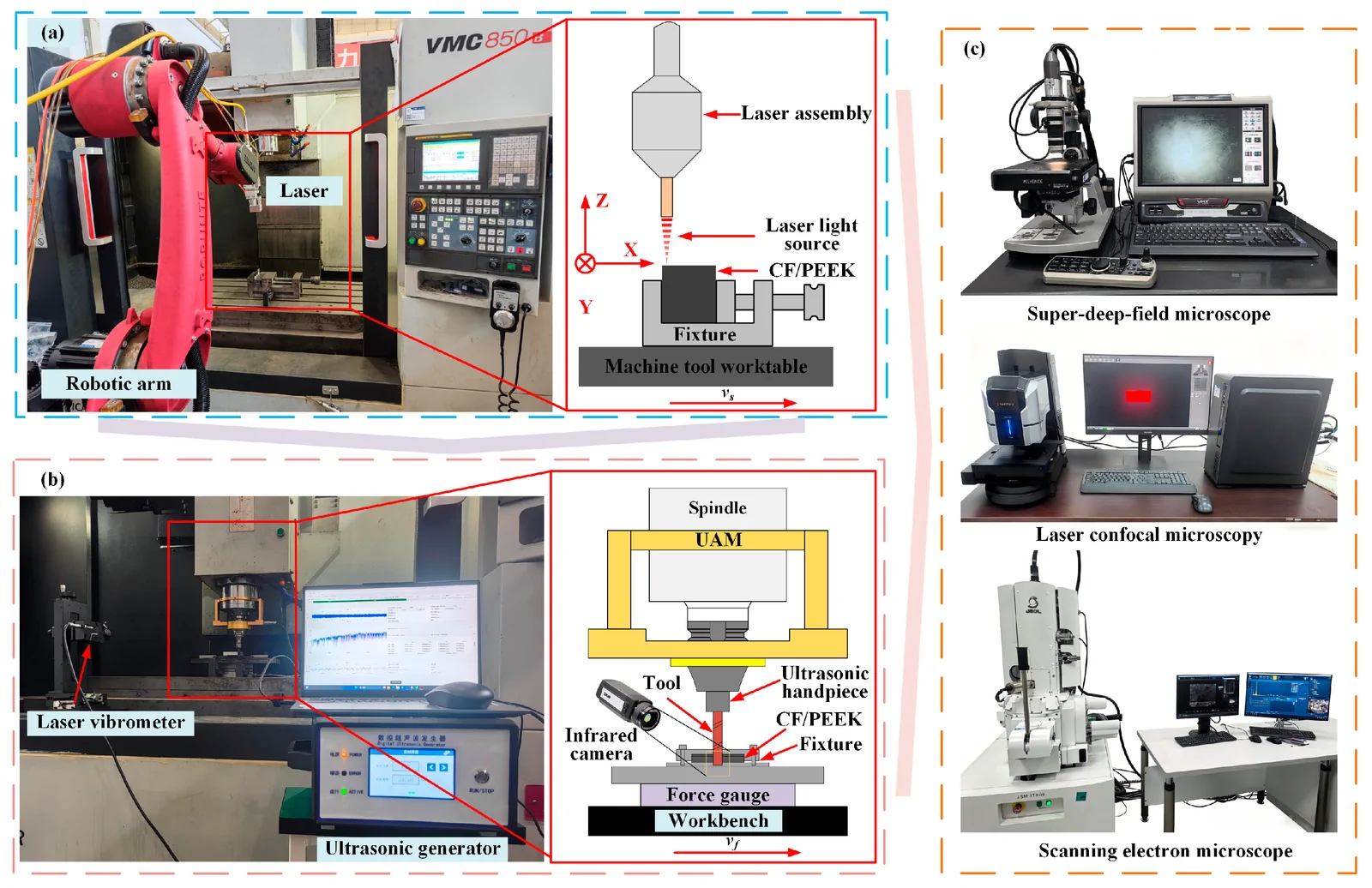
Experimental setup. (**a**) Laser processing; (**b**) ultrasonic vibration-assisted machining; (**c**) characterisation instruments.

A dedicated experimental setup was developed, as shown in Figure 4a,b. Laser irradiation and ultrasonic vibration-assisted milling were conducted sequentially rather than simultaneously. The process comprised laser scanning followed by ultrasonic vibration-assisted side milling, both conducted on a VMC 850B vertical machining centre.

The laser scanning system consisted of an RFL-QCW150/1500 quasi-continuous-wave fiber laser (Raycus) and a six-axis robotic arm (Figure 4a). The laser operated at a nominal wavelength of 1080 ± 5 nm. According to the manufacturer, the maximum peak power, maximum average pulsed power, repetition-frequency range, pulse-duration range, and maximum single-pulse energy are 1500 W, 150 W, 0–5000 Hz, 0.05–50 ms, and 15 J, respectively. These are manufacturer-rated specifications rather than the pulse parameters used in individual experiments. Pulse frequency, pulse duration, and duty cycle were fixed at the preset controller settings, while laser power and scanning speed were varied according to the experimental design. The beam-intensity profile was not measured experimentally, and a Gaussian distribution was therefore assumed in the theoretical analysis (Section 2.1). During scanning, the CF/PEEK laminate was mounted vertically with the laser beam normal to the predefined machining region. The surface spot radius was estimated using a graduated ruler to be approximately 1.5 mm; the focal position was not measured separately. Because the absorptivity of CF/PEEK at 1080 nm was not measured, the absolute absorbed laser energy could not be determined. Comparisons were therefore based on the prescribed incident laser parameters under consistent material and surface conditions.

After laser scanning, the heat-affected zone (HAZ) was examined using a VHX-2000C ultra-depth-of-field microscope (Figure 4c). Because HAZ characterization was conducted between the two stages, no fixed, short delay was imposed between laser scanning and ultrasonic milling. Following HAZ characterization, ultrasonic vibration-assisted milling was conducted within the laser-pretreated region under the prescribed cutting conditions.

As shown in Figure 4b, ultrasonic vibration-assisted milling was conducted on a vertical machining centre equipped with a BT40 ultrasonic tool holder. The vibration frequency and amplitude were adjusted using the ultrasonic generator. The apparent surface temperature was monitored using a FLIR T630sc infrared camera at 50 Hz (20 ms per frame), focused on the observable tool–workpiece interaction region with the CF/PEEK emissivity set to 0.95. The factory-calibrated camera was checked against a room-temperature reference before testing. The lens provided a field of view of ≥45° and an angular spatial resolution of ≤0.68 mrad. Because measurements were performed over a short distance under stable indoor conditions, no additional atmospheric correction was applied. Owing to the limited temporal resolution, individual tooth engagements and ultrasonic vibration cycles could not be resolved. Therefore, the recorded values represent the apparent surface-temperature response of the observable cutting region rather than the instantaneous maximum tool–workpiece interface temperature and were used primarily for comparison among machining conditions.

The cutting force was measured using a KISTLER 9257B dynamometer connected to a Type 5070A charge amplifier and a data acquisition unit. The measurement range was set to 0–500 N, and the sampling frequency was 1000 Hz. The force signals were acquired and directly exported using the default acquisition settings, without additional user-defined digital filtering. Before force extraction, baseline zeroing was performed, and the transient stages associated with tool entry and exit were excluded. A steady-state cutting interval was selected for analysis. After milling, surface topography was characterized using a KathMatic KC-X1000 laser spectral confocal microscope in full-resolution mode, with a vertical resolution of up to 2 nm and a laser spot diameter of approximately 2 μm. Each measurement covered an evaluation area of 500 μm × 500 μm, and three measurements were averaged for each specimen. Areal roughness *S_a_* was evaluated according to ISO 25178-2 after surface levelling to remove the overall tilt and Gaussian filtering (*λ_s_* = 2.5 μm, *λ_c_* = 0.08 mm) in accordance with ISO 16610-61. The same processing procedure was applied to all specimens. As shown in Figure 4c, representative line profiles were extracted from the three-dimensional topography solely to illustrate local surface-height variations. A JEOL JSM-IT 800 field-emission scanning electron microscope (SEM) was used to analyse damage on the machined surface. The SEM images were further processed using Fiji (ImageJ, version 1.54p) software to calculate the corresponding damage area. The equipment details are shown in Table 2.

#### 2.4.2. Experimental Design and Parameter Selection

A one-factor-at-a-time (OFAT) design was adopted to investigate the individual effects of laser power, scanning speed, ultrasonic vibration frequency, and amplitude on the machining responses of CF/PEEK laminates. Each factor was tested at three levels (Table 3), with the intermediate level used as the reference condition: 400 W laser power, 300 mm/min scanning speed, 34 kHz vibration frequency, and 5 μm amplitude. When one factor was varied, the other three were maintained at their reference values. Thus, nine unique parameter combinations were tested for each fiber orientation, as listed in Table 4, and the same matrix was applied to both 0° and 90° orientations. Each condition was repeated three times, resulting in 54 machining tests, and the mean of the three measurements was used for analysis. In the present study, machined surface quality was assessed specifically in terms of the areal roughness parameter *S_a_* and SEM-observed surface damage, whereas cutting force and infrared-measured apparent surface temperature were treated as machining responses that influence surface formation rather than as direct surface-quality indicators. The OFAT design was intended to identify individual parameter–response trends rather than parameter interactions or a globally optimal parameter combination.

The investigated parameter ranges were selected based on preliminary experiments, equipment operating characteristics, and previous studies. The spindle speed and feed rate were fixed at 6000 r/min and 480 mm/min, respectively, based on our previous results [26]. Laser power and scanning speed were selected to provide representative variations in laser energy input within the stable operating range. Under the investigated conditions, the surface heat-affected zone (HAZ) width was approximately 3–5.5 mm, measured perpendicular to the laser scanning direction, while the laser spot radius was approximately 1.5 mm. Accordingly, the width of cut was set to 7 mm to fully encompass the maximum observed HAZ. Ultrasonic vibration frequency and amplitude were selected within the reliably adjustable range of the system and were verified at the tool end face using a KV-HE4525L non-contact vibrometer before machining. The selected ranges were intended to reveal machining-response trends under practical operating conditions rather than to represent globally optimized process parameters.

## 3. Results and Discussion

### 3.1. Surface Roughness and Microscopic Surface Morphology

**Figure 5:**
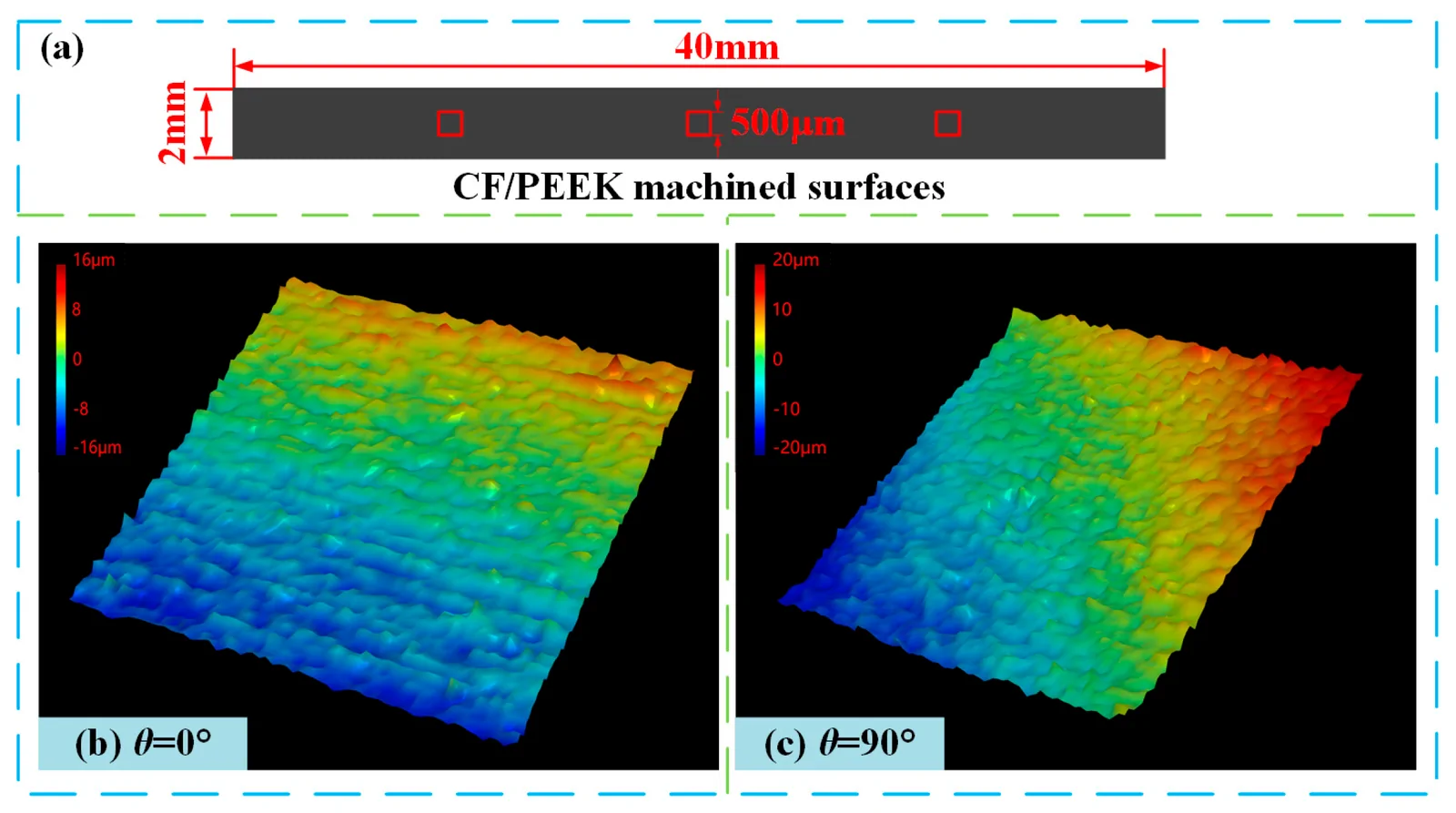
Schematic illustration of surface roughness measurement and machined surface morphologies of CF/PEEK unidirectional laminates: (**a**) schematic of the sampling regions on the machined surface of CF/PEEK; (**b**) microscopic morphology of the machined surface of CF/PEEK at *θ* = 0°; (**c**) microscopic morphology of the machined surface of CF/PEEK at *θ* = 90°.

In practical applications of CF/PEEK structural components, the quality of the machined surface is of critical importance. Surface roughness is a key parameter for evaluating the surface morphology of the workpiece [27]. As shown in Figure 5a, three regions of 500 μm × 500 μm were uniformly selected in the central part of the workpiece along the feed direction for measurement, and the average value was taken. Figure 5b,c shows the microscopic morphologies of the machined surfaces of specimens with 0° and 90° fiber orientations observed under the conditions of *P* = 400 W, *v*_s_ = 300 mm/min, *f* = 34 kHz. *A* = 5 μm. It can be seen that the machined surface of the specimen with the 0° fiber orientation exhibited relatively small fluctuations, continuous and strongly directional textures, and banded parallel features aligned with the feed direction, together with a low surface height variation. By contrast, the surface of the specimen with the 90° fiber orientation showed pronounced undulations, disordered and unevenly distributed textures, and irregular asperities, grooves and micropits, with weak directionality and a marked peak-to-valley difference.

**Figure 6:**
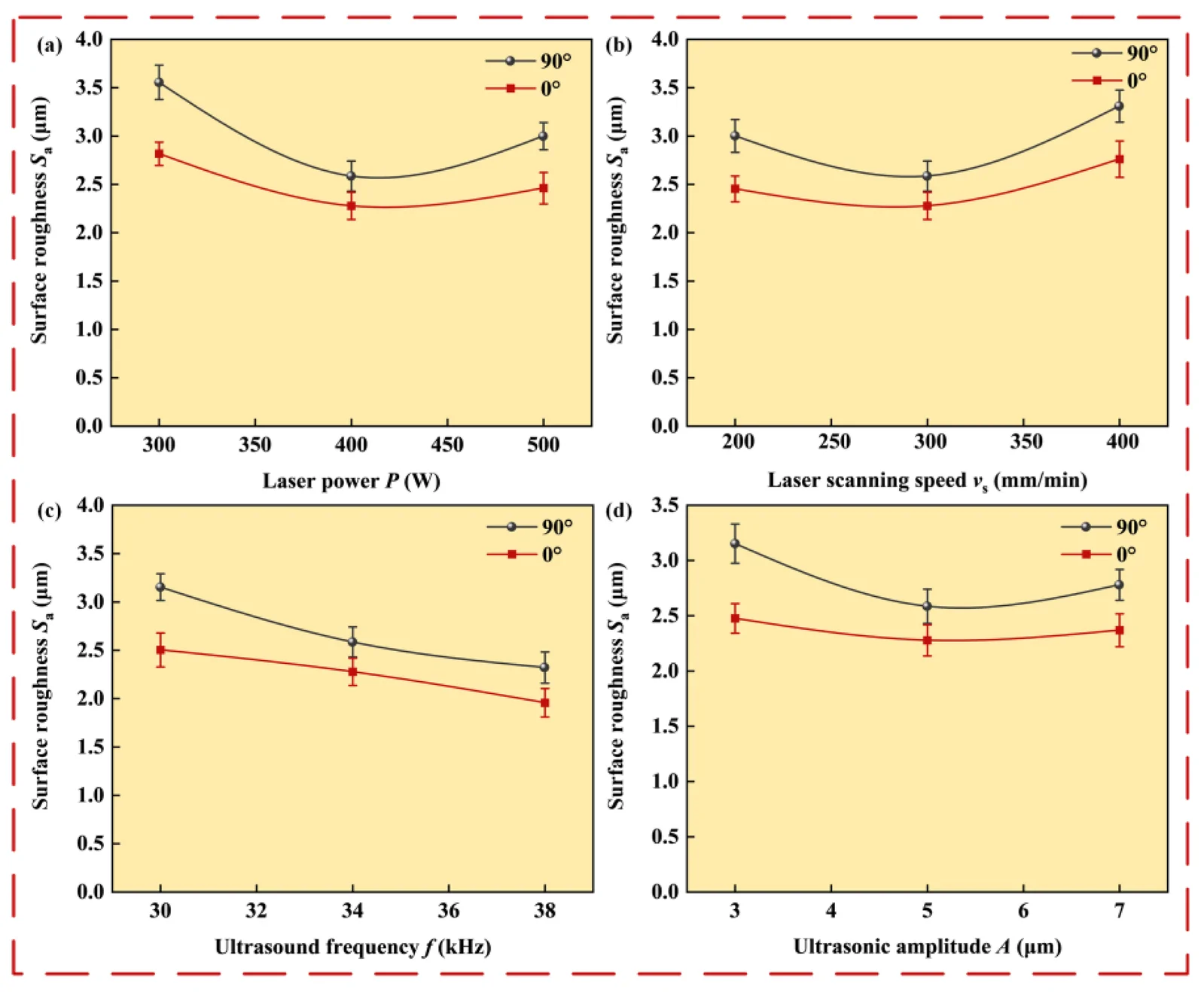
Effects of machining parameters on the surface roughness *S*_a_ of CF/PEEK unidirectional laminates. (**a**) *v*_s_ = 300 mm/min, *f* = 34 kHz, *A* = 5 μm; (**b**) *P* = 400 W, *f* = 34 kHz, *A* = 5 μm; (**c**) *P* = 400 W, *v*_s_ = 300 mm/min, *A* = 5 μm; (**d**) *P* = 400 W, *v*_s_ = 300 mm/min, *f* = 34 kHz.

Figure 6a shows that under all parameter conditions, the *S*_a_ value at the 90° fiber orientation was higher than that at 0°. This is mainly because, when the fiber orientation was 0°, the tool cut along the fiber direction and the cutting edge acted in a push-cutting manner, resulting in a lower cutting force, cleaner fiber shearing, a smoother surface, and consequently a lower Sa value. By contrast, at the 90° fiber orientation, the tool had to sever the fibers transversely, which significantly increased the cutting force and made the fibers more prone to bending and pull-out, thereby producing pits and fracture features on the surface and leading to a consistently higher *S*_a_ value.

When only the laser power was varied (Figure 6a), the surface roughness *S_a_* of CF/PEEK unidirectional laminates with 0° and 90° fiber orientations first decreased and then increased slightly with increasing laser power. A moderate laser power can sufficiently soften the PEEK matrix and thereby stabilize the cutting process; when the power is too low, the preheating is insufficient, whereas excessively high power causes matrix melting and increased hardness after resolidification, which in turn increases the cutting force. As shown in Figure 6b, with the other machining parameters held constant, the surface roughness *S*_a_ at both 0° and 90° fiber orientations exhibited a U-shaped dependence on the feed speed *v*_s_, reaching a minimum at approximately 300 mm/min; the underlying mechanism was similar to that associated with the variation in laser power.

The frequency dependence in Figure 6c differed: *S*_a_ decreased continuously at both fiber orientations with increasing ultrasonic frequency from 30 to 38 kHz, whereas the cutting force and infrared-derived surface temperature reached minima at 34 kHz before increasing or remaining stable at 38 kHz. This difference arises because force and temperature describe instantaneous mechanical and thermal responses, whereas *S*_a_ is governed by the resulting surface topography and machining-induced damage. At a constant amplitude, higher frequency introduces more vibration cycles per unit feed distance and may reduce the interval between successive tool–workpiece engagements, thereby refining surface marks and limiting fiber pull-out. However, the associated increases in vibration velocity and acceleration may enhance dynamic impact, local friction, and energy dissipation. Consequently, 34 kHz resulted in the lowest mechanical load and thermal response, whereas 38 kHz produced the minimum *S*_a_ value. Therefore, the optimal frequency is criterion-dependent and varies with the targeted performance metric.

When only the amplitude was varied (Figure 6d), the surface roughness *S_a_* at both 0° and 90° fiber orientations first decreased and then increased slightly with increasing amplitude. This trend can be attributed to the fact that a low amplitude provides insufficient intermittent cutting and friction reduction effects, whereas an excessively large amplitude intensifies the impact action and tends to induce local surface chipping.

**Figure 7:**
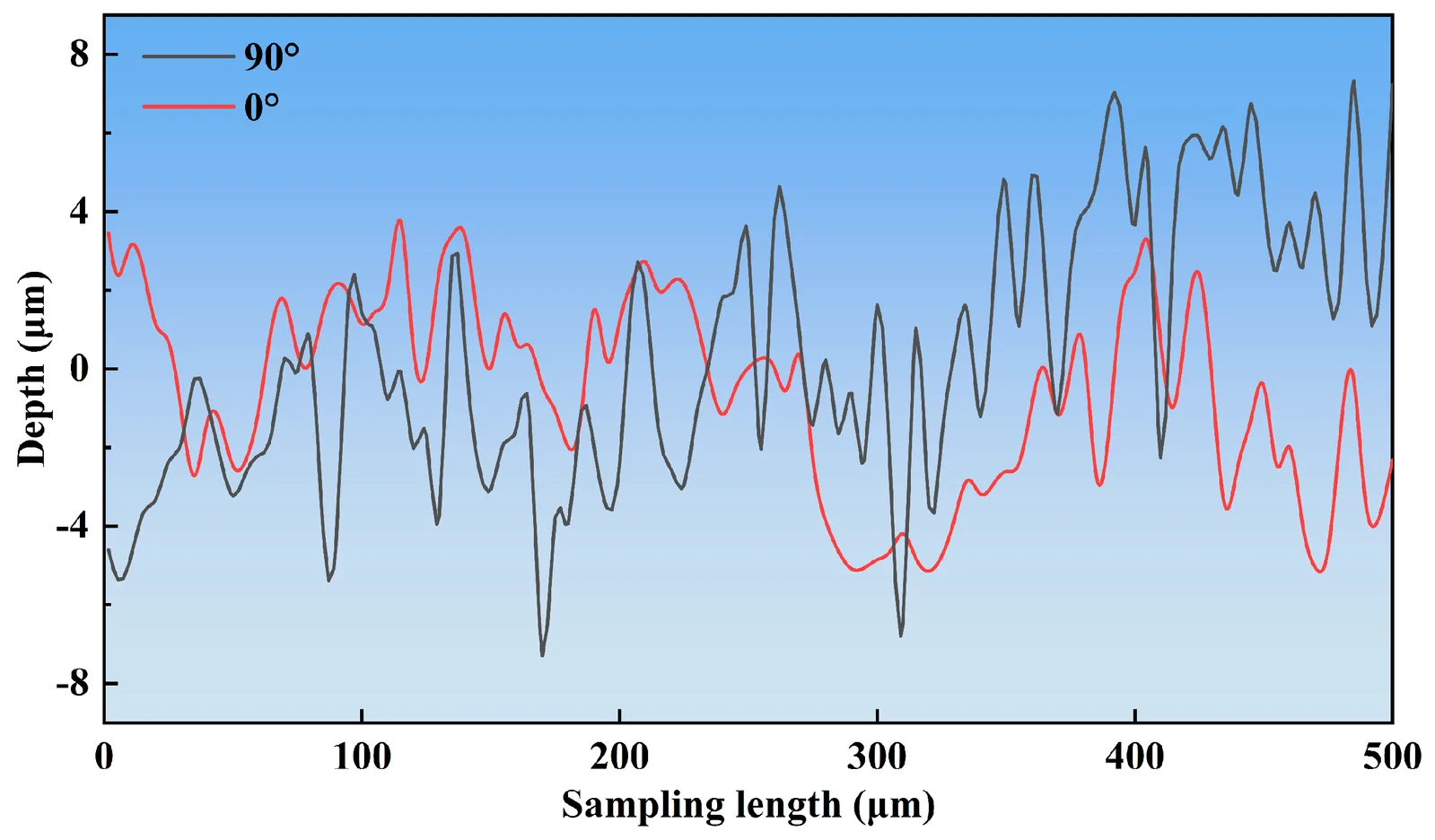
Representative surface height profiles extracted from the machined surfaces of CF/PEEK unidirectional laminates.

As shown in Figure 7, under the conditions of *P* = 400 W, *v*_s_ = 300 mm/min, *f* = 34 kHz and A = 5 μm, representative surface-height profiles were extracted from the centre of the 500 μm × 500 μm measurement region and compared. The profiles were extracted from the middle of the surface roughness measurement region to ensure the consistency and comparability of the measurements, with an evaluation length of 500 μm. It can be seen that the line roughness profile at the 0° fiber orientation was relatively uniform, with smaller surface undulations; by contrast, the profile at the 90° fiber orientation exhibited pronounced fluctuations and a larger undulation amplitude. This is mainly because, during milling, the carbon fibers at the 90° orientation are more prone to brittle fracture and fiber pull-out, producing pits and irregular surface features and thereby resulting in larger fluctuations in the roughness profile.

### 3.2. Machining-Induced Surface Defects

**Figure 8:**
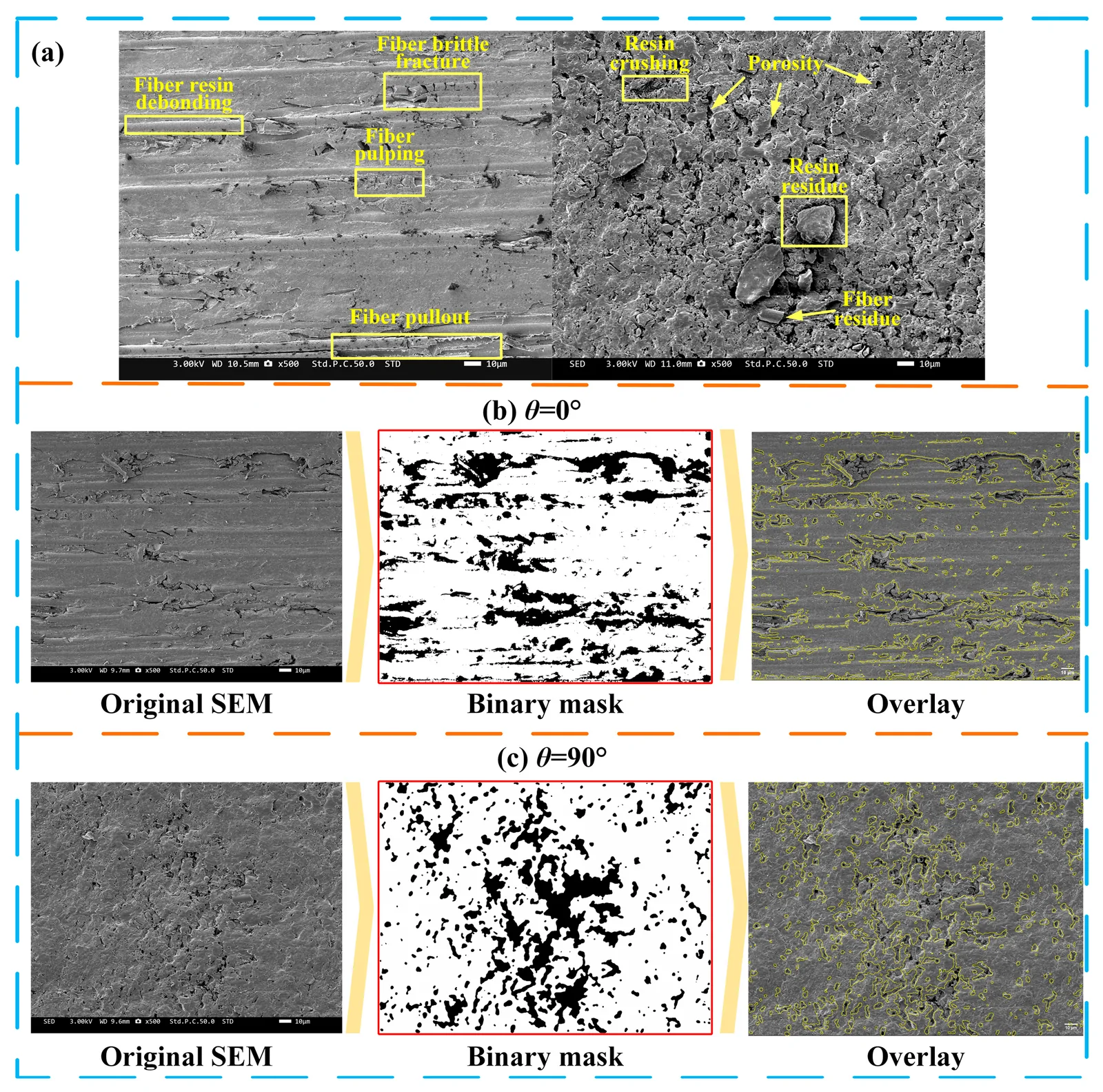
SEM-based identification of apparent surface damage in CF/PEEK laminates. (**a**) typical machining-induced surface features; (**b**) damage identification process for the 0° fiber orientation; (**c**) damage identification process for the 90° fiber orientation. Identical image-processing parameters were applied to all SEM images.

To quantitatively evaluate the apparent surface damage of machined CF/PEEK unidirectional laminates, specimens with nominal 0° and 90° fiber orientations were examined by scanning electron microscopy (SEM). Typical machining-induced features, including fiber pull-out, interfacial debonding, matrix fragmentation, and voids, are shown in Figure 8a. Uniform morphological criteria were applied to all SEM images to distinguish damage-related and intact regions. Fiber pull-out, interfacial debonding, matrix fragmentation/cracking, pronounced matrix smearing/redeposition, and open pores or cavities were classified as damage-related features. In contrast, intact exposed fibers without evident interfacial separation, regular machining grooves without cracking or material loss, and continuous matrix regions were classified as intact. Thus, exposed fibers and machining grooves were not considered damage per se. The same classification criteria were applied to both fiber orientations and all machining conditions.

For each specimen, three equally spaced SEM fields were acquired at 500× with a field of view of 256 μm × 196 μm and a resolution of 1280 × 960 pixels. Image processing was performed using Fiji (ImageJ, version 1.54p). After removal of scale bars and annotations, the images were converted to 8-bit grayscale and processed using the Auto Local Threshold–Phansalkar algorithm with a radius of 15 pixels and the default parameters (parameter 1 = 0.25 and parameter 2 = 0.50). The same settings were applied to all images without image-specific adjustment, followed by one binary opening and closing operation to suppress isolated noise and small discontinuities. Because SEM contrast may also be influenced by fiber/matrix contrast, surface inclination, charging, and imaging conditions, the thresholded regions were cross-checked against the corresponding original SEM images and overlays and were regarded as apparently damage-related regions rather than absolute physical damage areas. The SEM-derived apparent surface damage factor was defined as

(6) $$F_{d}=\frac{A_{\mathrm{seg}}}{A_{\mathrm{ROI}}}$$

where *A*_seg_ is the segmented area, and *A*_ROI_ is the analysed area. *F*_d_ is therefore used as a comparative semi-quantitative index. The sensitivity of *F*_d_ to the local thresholding scale was evaluated by varying the Phansalkar radius by ±18.6% (12, 15, and 18 pixels), while keeping the other parameters unchanged. The maximum relative variation in *F*_d_ was 7.2%, indicating limited sensitivity to the local thresholding scale. For each specimen, *F*_d_ was reported as the mean ± standard deviation of the three analysed SEM fields.

Figure 8b,c shows the original SEM images, binary segmentation masks, and corresponding overlays for the nominal 0° and 90° fiber orientations under *P* = 400 W, *v*_s_ = 300 mm/min, *f* = 34 kHz and *A* = 5 μm. For visualization, the binary masks were inverted so that the segmented apparent damage-related regions are displayed in black, while the yellow contours in the overlays indicate the corresponding segmentation boundaries. The original SEM images and corresponding overlays were used to cross-check the segmented regions according to the predefined morphological criteria. Identical classification criteria and image-processing parameters were applied to both fiber orientations and all machining conditions.

**Figure 9:**
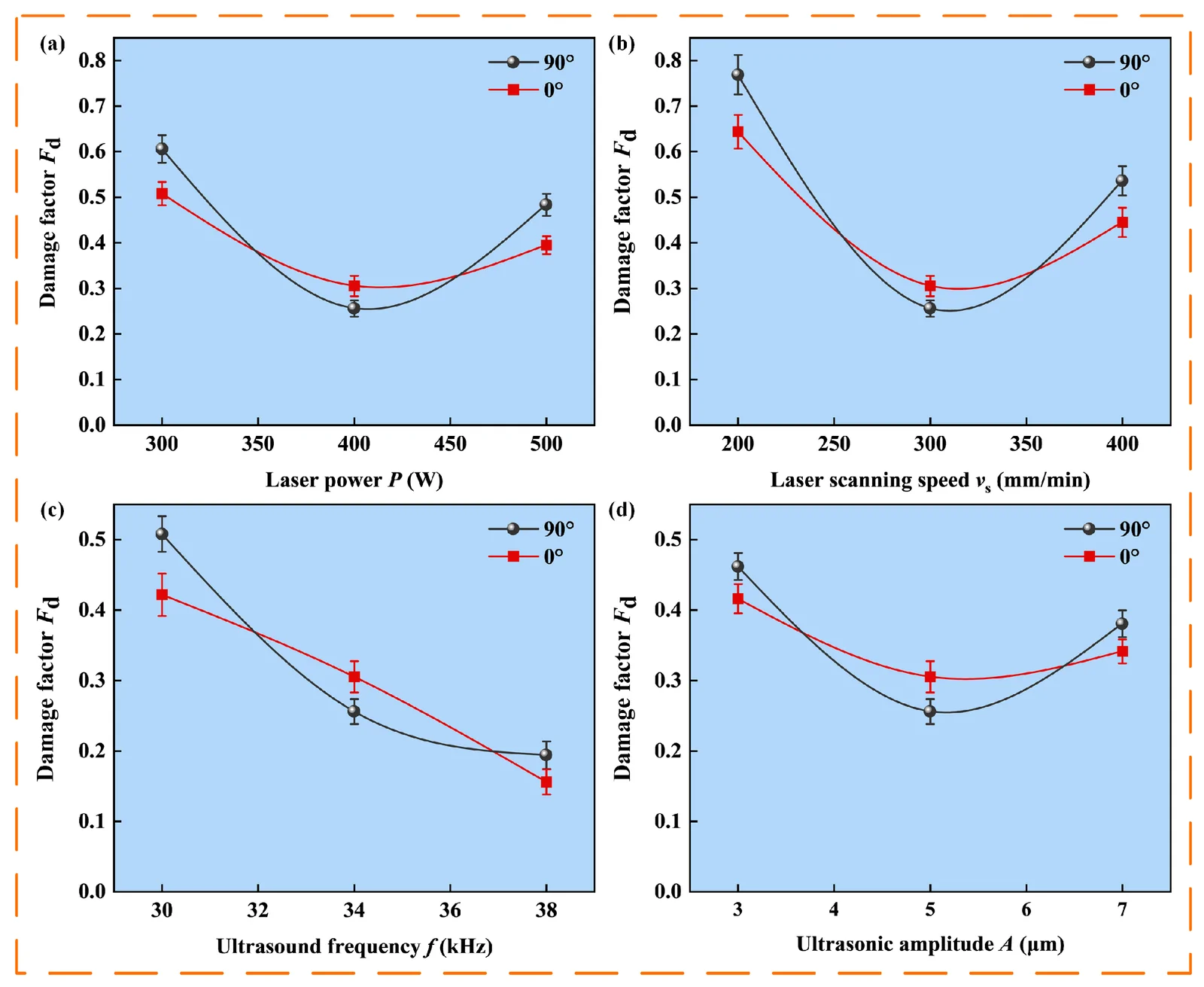
Variation in the surface damage factor *F*_d_ of CF/PEEK unidirectional laminates. (**a**) *v*_s_ = 300 mm/min, *f* = 34 kHz, *A* = 5 μm; (**b**) *P* = 400 W, *f* = 34 kHz, *A* = 5 μm; (**c**) *P* = 400 W, *v*_s_ = 300 mm/min, *A* = 5 μm; (**d**) *P* = 400 W, *v*_s_ = 300 mm/min, *f* = 34 kHz.

Figure 9 shows the apparent surface damage factor *F*_d_, quantified from SEM images, for surfaces machined at fiber orientations of 0° and 90° under different conditions. Across the tested conditions, *F*_d_ broadly followed the trend in surface roughness, although the two metrics capture distinct aspects of machined surface quality. At *P* = 400 W, *v_s_* = 300 mm/min, *f* = 34 kHz and *A* = 5 μm, *F*_d_ was 0.3054 ± 0.0223 at 0° and 0.2560 ± 0.0178 at 90°. Thus, *F*_d_ was approximately 19.3% higher at 0° than at 90°. A two-sided Welch’s t-test confirmed that this difference was statistically significant (*p* = 0.042, n = 3). This difference does not imply inconsistency between the two metrics. Surface roughness reflects variations in surface height, whereas *F*_d_ quantifies the area fraction of apparent damage features identified in SEM images. Widespread but shallow matrix smearing may therefore increase *F*_d_ without causing a comparable rise in surface roughness. Under these conditions, the 90° surface exhibited small, dispersed microvoids and shallow grooves. By contrast, matrix smearing, redeposition and mild plastic deformation were more pronounced at 0°. These matrix-dominated features may enlarge the apparent damage area detected from SEM images, thereby contributing to the higher *F*_d_ observed at 0°.

### 3.3. Cutting Force Characteristics

**Figure 10:**
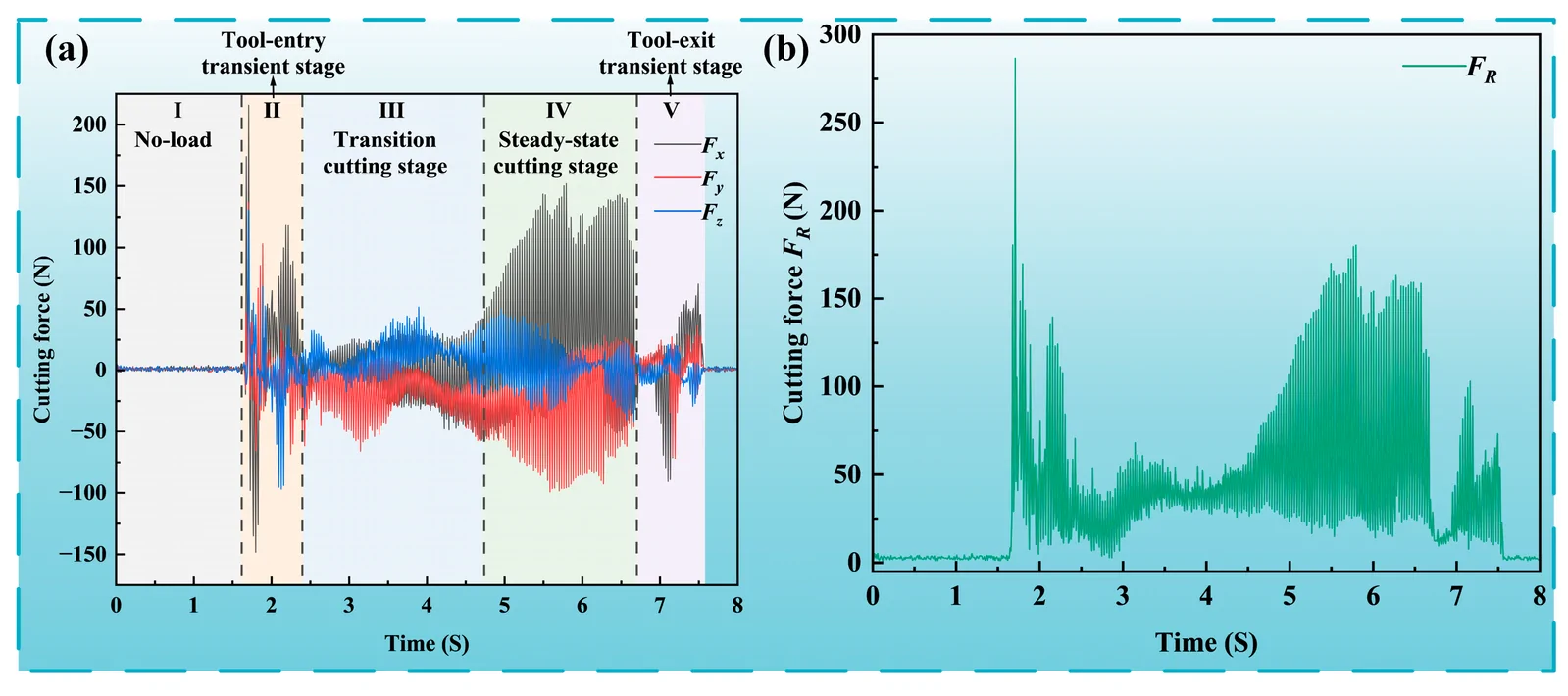
Time-domain cutting force signals at a fiber orientation angle of 90°. (**a**) three orthogonal force components *F_x_*, *F_y_*, and *F_z_*; (**b**) resultant cutting force *F_R_*.

In the machining of CF/PEEK composites, Cutting force is an important machining response, and excessive cutting force may promote machining-induced damage, as excessive forces may lead to severe machining damage [28]. In this study, the feed, radial, and axial directions were defined as the X-, Y-, and Z-axes, respectively, with the corresponding force components denoted as *F_x_*, *F_y_*, and *F_z_*. The resultant cutting force was calculated as $F_{R}\;=\;{\left(F_{x}^{2}\;+\;F_{y}^{2}\;+\;F_{z}^{2}\right)}^{1/2}$. Figure 10a presents representative time-domain signals of *F_x_*, *F_y_*, and *F_z_* at a fiber orientation angle of 90° under the conditions of *P* = 400 W, *v_s_* = 300 mm/min, *f* = 34 kHz, and *A* = 5 μm. The force signal was divided into five characteristic stages: Region I, the no-load stage; Region II, the tool-entry transient stage; Region III, the transition cutting stage; Region IV, the steady-state cutting stage; and Region V, the tool-exit transient stage. The corresponding resultant cutting force signal *F_R_* is shown in Figure 10b. Since *F_x_* is aligned with the feed direction and directly reflects the cutting resistance associated with material removal, it was selected as the primary parameter for quantitative comparison. For each cutting condition, the local peak values of *F_x_* over multiple cutting cycles within the steady-state cutting interval (Region IV) were identified, and their mean value was taken as the representative cutting force. The variations in *F_y_*, *F_z_*, and *F_R_* were also examined to characterize the overall force response. Although the three force components exhibited different magnitudes, *F_x_*, *F_y_*, and *F_z_* varied consistently across the cutting stages, indicating the three-dimensional and intermittent cutting characteristics. During steady-state cutting, the resultant force *F_R_* exhibited periodic fluctuations. Considering its direct physical correspondence with the feed-direction cutting resistance, *F_x_* was selected as the primary parameter for subsequent quantitative analysis.

**Figure 11:**
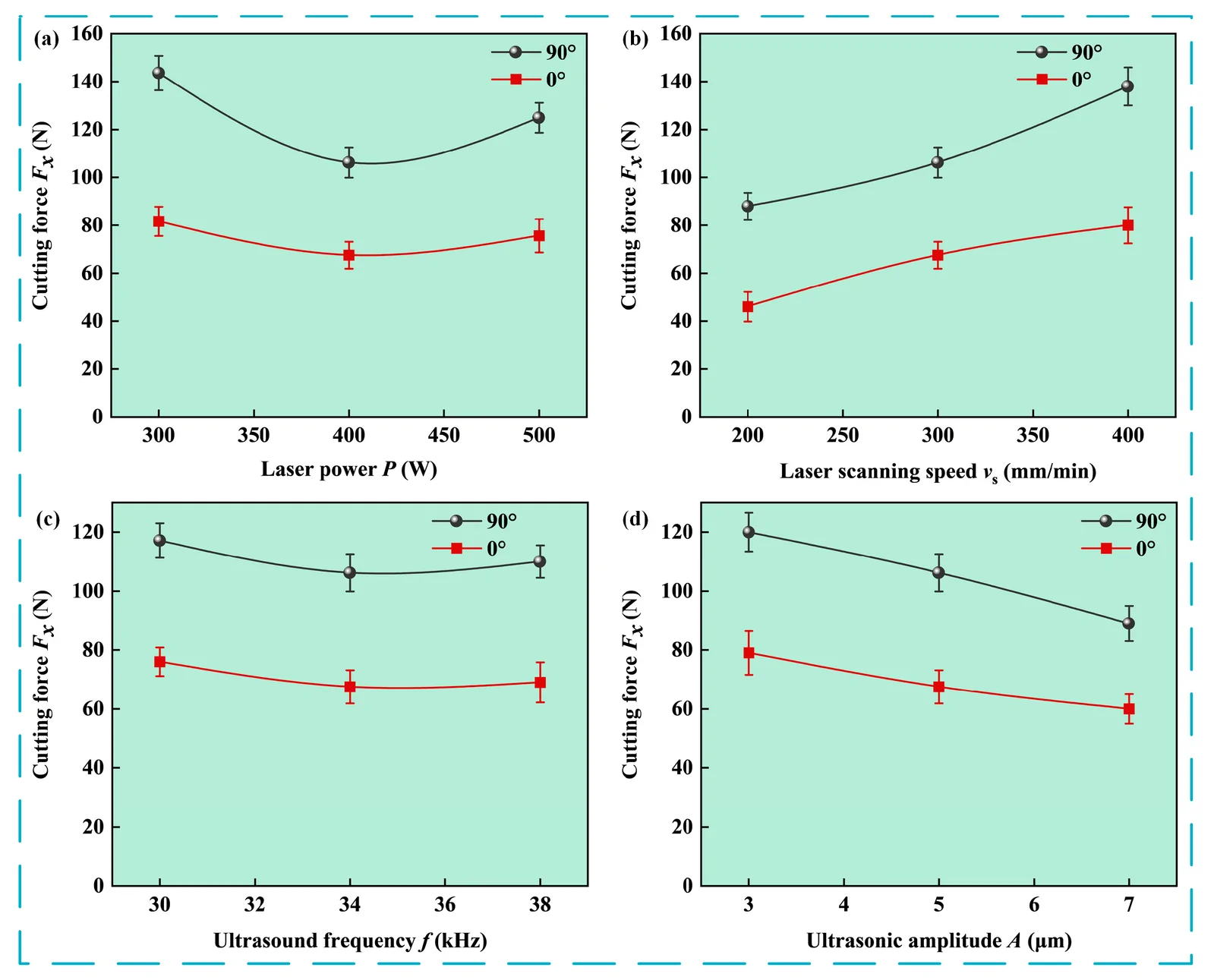
Variation in cutting force for CF/PEEK unidirectional laminates. (**a**) *v*_s_ = 300 mm/min, *f* = 34 kHz, *A* = 5 μm; (**b**) *P* = 400 W, *f* = 34 kHz, *A* = 5 μm; (**c**) *P* = 400 W, *v*_s_ = 300 mm/min, *A* = 5 μm; (**d**) *P* = 400 W, *v*_s_ = 300 mm/min, *f* = 34 kHz.

Figure 11 shows the effects of different machining parameters on the cutting force component *F_x_* at fiber orientations of 0° and 90°. *F_x_* at the 90° fiber orientation was consistently higher than that at 0° under all machining conditions. This can mainly be attributed to the fact that, under 90° milling, the tool must cut directly across the carbon-fiber bundles, accompanied by greater transverse fiber-cutting resistance and stronger fiber–matrix interaction; by contrast, during milling at 0°, material removal occurs predominantly through shearing and pull-out along the fiber direction, resulting in a significantly lower cutting resistance.

With all other parameters kept constant, Figure 11a shows the effect of laser power on the cutting force at fiber orientations of 0° and 90°. As the laser power increased from 300 W to 400 W, *F_x_* decreased markedly; when the power was further increased to 500 W, *F_x_* rose slightly. Overall, the cutting force was highest at 300 W, lowest at 400 W, and intermediate at 500 W, indicating that an optimal laser-power window exists for minimising the cutting force. This can be attributed to the fact that, at 300 W, the thermal input was insufficient to effectively remove or soften the PEEK matrix, so the tool had to rely on a greater mechanical load to sever the fibers and disrupt the interface, resulting in the highest *F_x_*. When the power increased to 400 W, the laser energy was sufficient to remove part of the matrix and markedly soften the PEEK within the heat-affected zone, thereby reducing *F_x_* to its minimum. However, when the laser power reached 500 W, the excessive energy caused rapid carbonisation of the PEEK matrix and formed a brittle carbonised layer on the surface, shifting the material from a thermally softened machinable state to a hard, brittle residue, which in turn led to a rebound in cutting force. At the 0° fiber orientation, *F_x_* decreased by 17.3% and 7.4% at 400 and 500 W, respectively, relative to 300 W; at 90°, the corresponding reductions were 26.1% and 12.9%. This difference is mainly attributed to the fact that, during milling at 0°, the tool cuts along the fiber direction, where the resistance is intrinsically lower; although changes in laser power affect the extent of matrix softening, their contribution to the overall mechanical response remains limited. By contrast, under 90° milling, the tool must cut directly across the fiber bundles, so the cutting force is inherently higher and much more sensitive to the supporting effect of the matrix. Therefore, when the laser power increased from 300 W to 400 W, matrix softening likely reduced the support and constraint provided by the matrix to the fibers, thereby facilitating fiber fracture, leading to a more pronounced reduction in cutting force.

As shown in Figure 11b, the cutting force increased with increasing laser scanning speed at both 0° and 90° fiber orientations. At the 0° fiber orientation, *F_x_* increased by 46.7% and 73.9%, respectively, whereas at 90°, the corresponding increases were 20.9% and 57.1%. Within the scanning-speed range of 300–400 mm/min, the increase in *F_x_* was relatively gradual at 0°, but markedly steeper at 90°. As the laser scanning speed increased, the laser preheating time was shortened and the degree of matrix softening was reduced, leading to an increase in cutting force at both fiber orientations. Because milling at 0° is dominated by matrix shearing and fiber pull-out, it is highly sensitive to matrix softening; accordingly, the increase in cutting force becomes more pronounced as the scanning speed rises. By contrast, milling at 90° is governed primarily by direct fiber fracture, and laser preheating has only a limited effect on fiber strength, resulting in a smaller increase in *F_x_*. However, within the range of 300–400 mm/min, matrix softening and the associated reduction in fiber support become insufficient, causing the cutting mode to shift from thermally softened failure to hard cutting, which makes the increase in *F_x_* steeper; by contrast, during 0° milling, a certain degree of matrix softening is still retained, so the increase remains relatively moderate.

Figure 11c shows the variation in *F_x_* with ultrasonic excitation frequency at fiber orientations of 0° and 90°. A non-monotonic variation in cutting force was observed at both fiber orientations. As the excitation frequency increased from 30 to 34 kHz, *F_x_* decreased, whereas a further increase to 38 kHz resulted in a slight increase. At the 0° fiber orientation, *F_x_* at 34 and 38 kHz was 11.2% and 9.2% lower, respectively, than that at 30 kHz. At the 90° fiber orientation, the corresponding reductions were 9.4% and 6.1%, respectively. Therefore, 34 kHz produced the minimum cutting force among the investigated excitation frequencies under the present machining conditions. The actual axial vibration amplitude of the tool was measured at each excitation frequency under the same nominal amplitude setting of 5 μm. The measured amplitudes at 30, 34, and 38 kHz were 4.891, 5.171, and 5.013 μm, respectively, corresponding to deviations of −2.18%, +3.42%, and +0.26% from the nominal setting. The measured amplitudes therefore remained close to the nominal input value over the investigated frequency range. Based on the cutting force results, 34 kHz produced the minimum *F_x_* among the investigated excitation frequencies under the present machining conditions. Moreover, the ultrasonic amplitude directly determines the contact characteristics and frictional behaviour at the tool–workpiece interface. As the amplitude increases, the instantaneous separation interval between the tool and the workpiece is prolonged, the effective contact duty cycle decreases, and interfacial adhesion and friction are periodically weakened, thereby significantly reducing the average cutting force; this trend is consistent with Figure 11d. At the 0° fiber orientation, the reductions in *F_x_* were 14.62% and 24.1%, respectively; at 90°, the corresponding reductions were 11.5% and 25.8%.

### 3.4. Infrared-Measured Apparent Surface Temperature

PEEK is a typical thermally sensitive polymer, and its mechanical properties deteriorate significantly when the local temperature in the cutting region approaches or exceeds its glass-transition temperature. This may lead to severe mechanical and thermal damage, including matrix degradation, material smearing, fiber pull-out, and void formation. Therefore, controlling the thermal response during the milling of CF/PEEK composites is important for optimizing the machining parameters.

**Figure 12:**
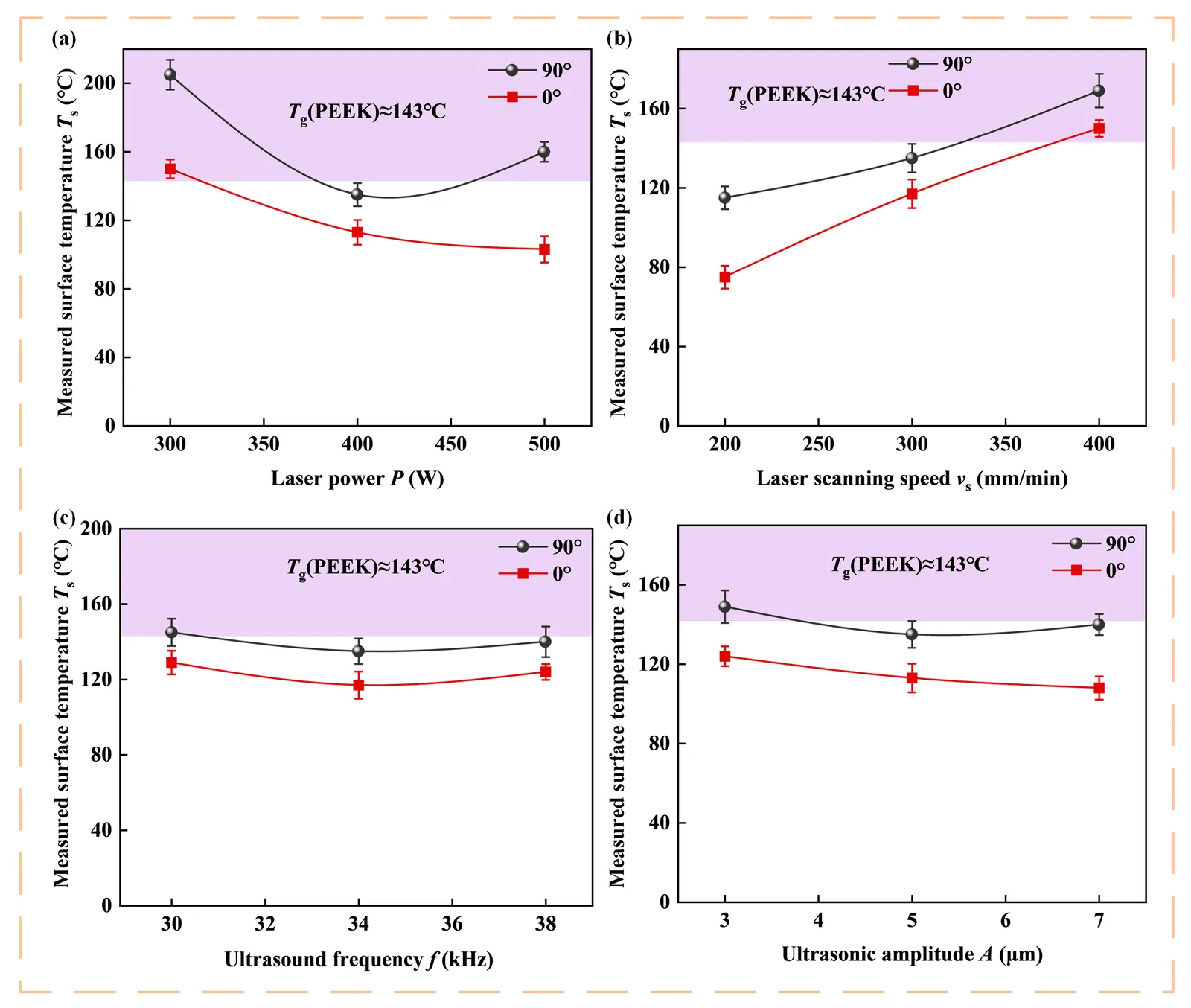
Variation in measured surface temperature for CF/PEEK unidirectional laminates. (**a**) *v*_s_ = 300 mm/min, *f* = 34 kHz, *A* = 5 μm; (**b**) *P* = 400 W, *f* = 34 kHz, *A* = 5 μm; (**c**) *P* = 400 W, *v*_s_ = 300 mm/min, *A* = 5 μm; (**d**) *P* = 400 W, *v*_s_ = 300 mm/min, *f* = 34 kHz. **Note:** The light-purple background indicates temperatures above the glass transition temperature of PEEK (*T_g_* ≈ 143 °C).

Figure 12 compares the infrared-measured surface temperatures in the observable cutting region of CF/PEEK at fiber orientations of 0° and 90° under different machining parameters. Under identical machining conditions, the measured surface temperature at the 0° fiber orientation was markedly lower than that at 90°, consistent with the trend observed for the cutting force. This difference is mainly attributed to the anisotropic thermomechanical characteristics of CF/PEEK composites. Carbon fibers exhibit much higher thermal conductivity along the fiber axis than in the transverse direction, whereas the PEEK matrix has relatively low thermal conductivity. When the milling direction is parallel to the fiber orientation, heat can be conducted more efficiently along the fiber axis and dissipated away from the observable cutting region, thereby reducing local surface heat accumulation. By contrast, when the milling direction is perpendicular to the fibers, heat transfer occurs mainly through the fiber transverse direction and the matrix; therefore, heat dissipation is restricted and local surface heat accumulation is more likely.

In addition, milling at 90° requires repeated transverse fiber fracture and is associated with stronger local fiber–matrix deformation and friction, which may generate greater mechanical dissipation and heat. Milling at 0° is characterized mainly by fiber shearing and pull-out and exhibits a relatively stable cutting process, resulting in less frictional heat generation. These mechanisms may explain why the infrared-measured apparent surface temperature was lower at 0° than at 90°. It should be emphasized that the infrared camera records the apparent surface temperature of the visible cutting region rather than the actual instantaneous tool–chip interface temperature.

Figure 12a shows that, within the investigated range, the measured surface temperature at the 0° fiber orientation decreased with increasing laser power and then gradually stabilized, whereas that at 90° first decreased and then increased, reaching a minimum at a laser power of approximately 400 W. Under this condition, the measured apparent surface temperature remained below the glass-transition temperature of PEEK. However, this observation does not demonstrate that the actual tool–chip interface temperature remained below the glass-transition temperature, because localized and transient interface temperatures may be higher than those detected by infrared thermography. An appropriate laser power may partially soften the PEEK matrix, thereby reducing friction and cutting force and consequently decreasing heat generation in the observable cutting region. This effect was particularly evident at 90°, whereas excessively high laser power increased the cutting force and was accompanied by an increase in the measured surface temperature. At 0°, increasing laser power produced a smaller temperature variation, possibly because heat was more readily conducted along the fibers. With increasing laser scanning speed (Figure 12b), the measured surface temperature at both fiber orientations increased. A higher scanning speed shortened the laser–material interaction time and reduced the extent of laser-induced thermal modification, which was accompanied by increased cutting force and consequently a higher apparent surface temperature in the observable cutting region.

When only the ultrasonic frequency was varied (Figure 12c), the measured surface temperatures at both 0° and 90° exhibited a U-shaped dependence on frequency, reaching their minimum values at 34 kHz. The temperature measured at 90° was more sensitive to frequency, and its reduction at 34 kHz relative to those at 30 and 38 kHz was markedly greater than that at 0°. This result suggests that, within the investigated frequency range, ultrasonic vibration at 34 kHz provided a more favorable dynamic cutting condition. It may have enhanced intermittent separation between the tool and workpiece, shortened the effective contact duration, and reduced interfacial friction and adhesion, thereby decreasing heat generation. At 0°, heat was more readily dissipated along the fibers, resulting in a weaker dependence of the measured surface temperature on ultrasonic frequency. Regarding the effect of ultrasonic amplitude (Figure 12d), the measured surface temperature at the 0° fiber orientation decreased markedly with increasing amplitude and then gradually approached a plateau. Heat conduction along the fiber axis may have promoted heat dissipation, causing the surface temperature to become less sensitive to further increases in amplitude. At 90°, the measured surface temperature initially decreased and then increased slightly, exhibiting an overall U-shaped trend with a minimum at approximately 5 μm. Because transverse heat conduction is restricted and the cutting process is dominated by transverse fiber fracture and interfacial sliding, an excessive amplitude may increase the impact velocity and lateral rubbing, thereby increasing local adhesive and plastic-dissipation heating. Within the limitations of the infrared measurement, these results indicate that increasing ultrasonic amplitude can generally reduce the apparent surface temperature in the observable cutting region. An amplitude of approximately 5 μm produced the minimum measured temperature at 90°, whereas the temperature at 0° decreased and then approached a plateau.

### 3.5. Thermomechanical Coupling and Material-Removal Mechanism

**Figure 13:**
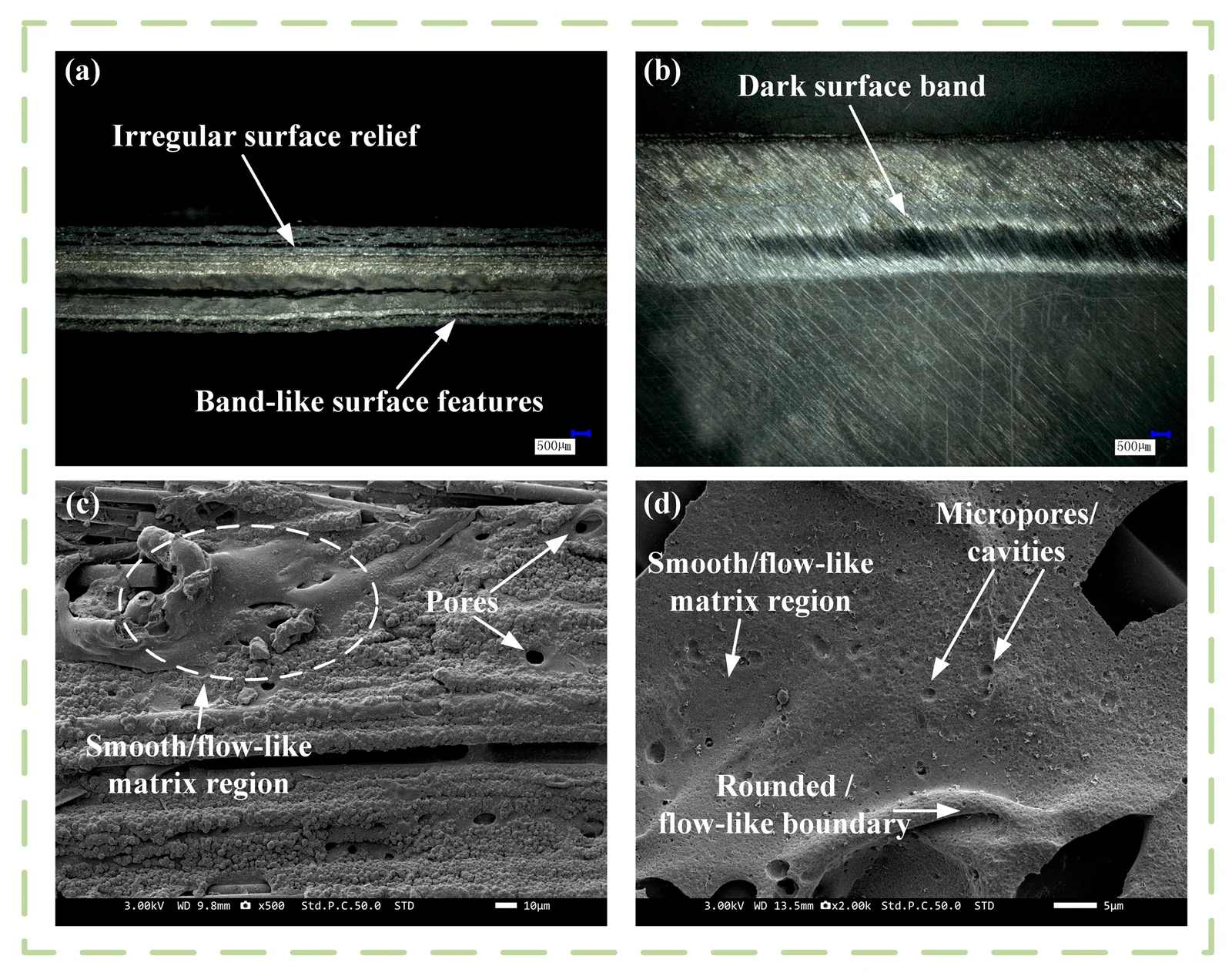
Multiscale morphology of CF/PEEK. (**a**) optical micrograph of the interlaminar region; (**b**) optical micrograph of the surface; (**c**) SEM overview (×500); (**d**) high-magnification SEM image (×2000).

Figure 13 presents optical and SEM observations of the laser-pretreated CF/PEEK surface under *P* = 500 W and *v_s_* = 300 mm/min Optical images reveal irregular band-like surface undulations and locally darkened regions within the laser-irradiated area (Figure 13a,b). At ×500 magnification, SEM images show granular deposits, smooth matrix regions, and voids on the treated surface (Figure 13c). At higher magnification (×2000), continuous smooth matrix regions with rounded boundaries and flow-like transitions are observed, together with pits and small voids (Figure 13d). The coexistence of smooth regions, rounded boundaries, granular deposits, and voids suggests that laser heating modified the local matrix morphology. Unlike the sharp fracture features associated with mechanical damage, these morphologies are more indicative of matrix deformation, migration, and redistribution under thermal loading. During laser pretreatment, the high thermal input may induce PEEK matrix softening, followed by possible local melting and resolidification. These thermal-induced modifications may alter the matrix support around carbon fibers and the fiber–matrix constraint conditions, thereby affecting subsequent fiber breakage, material removal during ultrasonic vibration-assisted milling.

**Figure 14:**
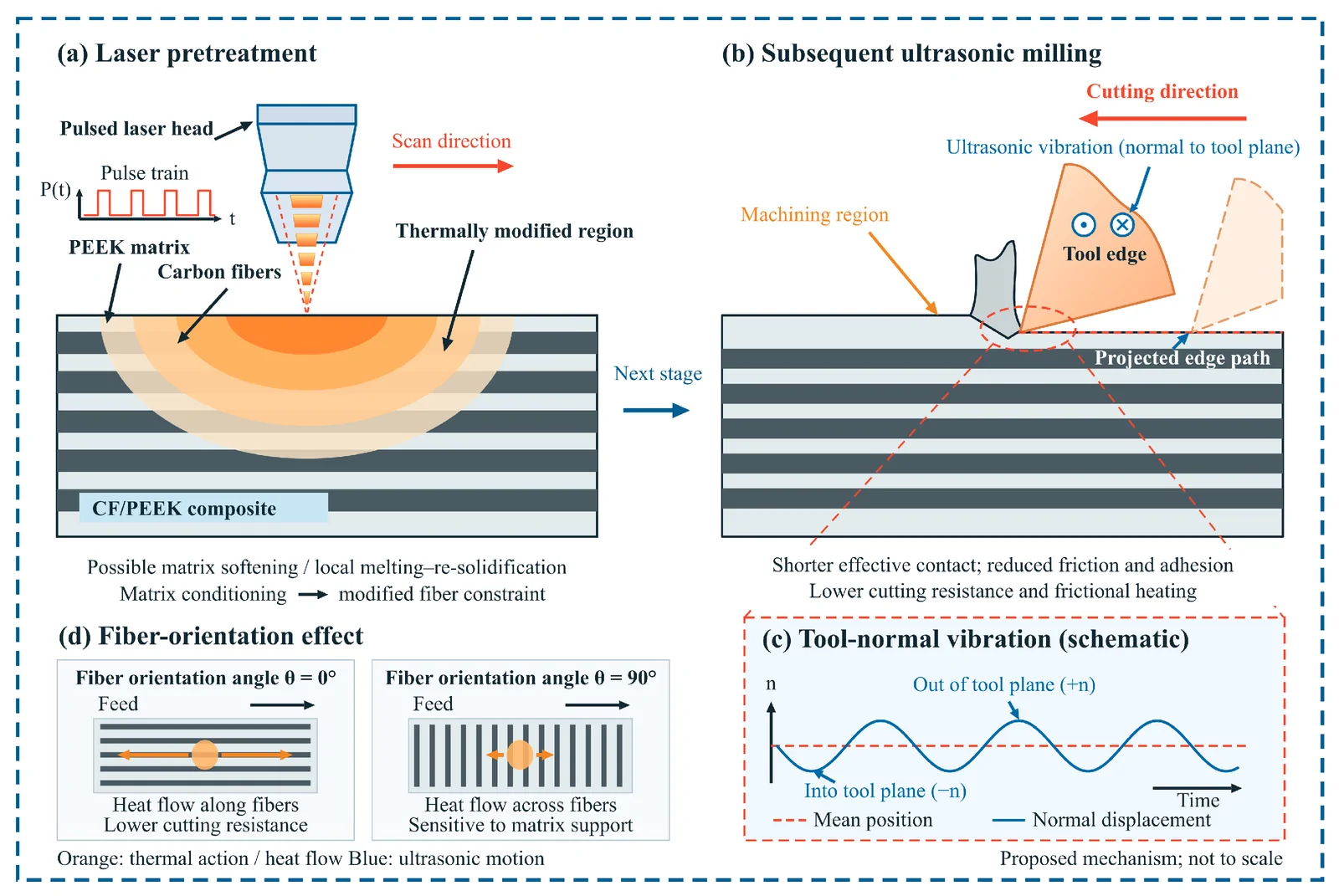
Proposed sequential thermomechanical mechanism of pulsed laser–ultrasonic-assisted milling of CF/PEEK: (**a**) pulsed laser pretreatment; (**b**) subsequent ultrasonic-assisted milling; (**c**) ultrasonic vibration normal to the tool plane; and (**d**) effects of fiber orientation. Note: The orange-to-yellow shaded areas schematically represent the thermally modified regions induced by pulsed laser irradiation and do not indicate quantitative temperature values. The symbols “⊙” and “⊗” indicate the ultrasonic vibration directions out of and into the tool plane, respectively.

The proposed machining mechanism involves a sequential thermomechanical process (Figure 14). Within a suitable thermal-input range, pulsed laser pretreatment may induce local softening and redistribution of the PEEK matrix, modifying its constraint on the carbon fibers and facilitating subsequent material removal. During ultrasonic-assisted milling, periodic vibration normal to the tool plane modulates local tool–workpiece contact, potentially reducing effective contact duration, friction and adhesion. Excessive vibration amplitude, however, may increase impact loading and local chipping. Together, laser-induced matrix modification and vibration-induced contact modulation may reduce cutting resistance and frictional heat generation, thereby influencing surface formation and damage.

The material response also depends on the fiber orientation angle. At 0°, removal predominantly follows the fiber direction, while preferential heat conduction along the fibers favors heat dissipation. These characteristics are consistent with the comparatively lower cutting forces and apparent surface temperatures. At 90°, transverse fiber cutting increases removal resistance and dependence on matrix support, while heat conduction across the fibers is more restricted. Consequently, the mechanical and thermal responses at 90° may be more sensitive to changes in the laser-modified matrix condition and ultrasonic contact behavior.

## 4. Conclusions

Laser-assisted ultrasonic vibration milling experiments were conducted on CF/PEEK unidirectional laminates with fiber orientations of 0° and 90°. The effects of laser power, laser scanning speed, ultrasonic vibration frequency, and vibration amplitude on cutting force, measured surface temperature, surface roughness, and surface damage were experimentally investigated. Based on the experimental results obtained within the investigated parameter ranges, the main conclusions are as follows:

(1) The machining parameters had pronounced effects on the cutting forces and temperatures of CF/PEEK composites. The 90° fiber orientation generally exhibited higher cutting force and temperature than the 0° orientation. This difference may be associated with the different material-removal behaviors caused by fiber orientation, including fiber-bundle fracture and tool–material interaction during transverse cutting. Increasing the laser power from 300 to 400 W reduced the cutting force by 7.4–17.3% and 12.9–26.1% for the 0° and 90° fiber orientations, respectively, whereas a further increase in laser power to 500 W resulted in a slight increase in cutting force. These results indicate that excessive laser power does not necessarily lead to a further reduction in machining load.

(2) Laser scanning speed and ultrasonic vibration parameters significantly influenced the machining responses of CF/PEEK composites. Increasing the laser scanning speed resulted in increases in both cutting force and measured surface temperature within the investigated range. The introduction of ultrasonic vibration was associated with a reduction in cutting force of 9.4–25.8% under the tested conditions, and the lowest cutting force among the investigated frequencies was obtained at 34 kHz. For the 90° fiber orientation, a vibration amplitude of approximately 5 μm produced the lowest measured surface temperature within the investigated amplitude range. These results demonstrate the sensitivity of the machining responses to ultrasonic vibration parameters; however, the underlying mechanisms require further direct experimental verification.

(3) Fiber orientation and processing parameters had pronounced effects on the machined surface quality. The 0° fiber orientation generally produced smoother and more continuous machined surfaces with fewer observable defects, whereas the 90° orientation exhibited more pronounced surface undulation, fiber pull-out, fiber fracture, and surface-observed fiber–matrix separation. Within the investigated parameter ranges, moderate laser power and scanning speed were associated with lower surface roughness and fewer surface defects. These trends may be related to laser-induced changes in the thermomechanical response of the PEEK matrix and the resulting tool–material interaction, but additional thermal and microstructural characterization is required to verify this interpretation.

(4) SEM observations revealed different degrees of fiber fracture, fiber pull-out, matrix-related defects, and surface-observed fiber–matrix separation under different processing conditions. The surface damage factor derived from SEM images exhibited a trend generally consistent with that of surface roughness, indicating that the two indicators provide complementary information for evaluating the machined surface quality of CF/PEEK laminates. The experimental results further showed that appropriate adjustment of laser and ultrasonic parameters can effectively regulate the machining responses and surface quality within the investigated ranges.

From an academic perspective, this study clarifies the fiber-orientation-dependent machining behaviour of CF/PEEK laminates under combined laser and ultrasonic assistance and establishes quantitative relationships between key process parameters and machining responses, including cutting force, temperature, surface roughness, and surface damage. These results provide a basis for further investigation of the thermomechanical interactions governing anisotropic composite removal.

From an engineering perspective, the observed parameter–response relationships provide preliminary guidance for process selection. Within the investigated ranges, 400 W laser power and 34 kHz vibration frequency produced relatively low cutting forces, while an amplitude of 5 μm resulted in the lowest measured surface temperature for the 90° fiber orientation. However, because a single-factor experimental design was used, these conditions should not be regarded as a globally optimal parameter combination, and laser–ultrasonic interactions cannot be fully resolved. In addition, damage characterization was limited to the exposed machined surface; subsurface damage, internal fiber–matrix debonding, and interlaminar delamination were not evaluated. Future work will therefore incorporate factorial designs and subsurface characterization to further clarify parameter interactions and determine whether the observed improvements extend beneath the machined surface.

## Figures and Tables

**Table 1:** Mechanical properties of the CF/PEEK composite.

Material	Density (g/cm^3^)	Tensile Strength (MPa)	Tensile Modulus (GPa)	Bending Strength (MPa)
CF/PEEK	1.57	2200	125	1800

**Table 2:** Main experimental equipment and instruments used in this study.

Equipment	Model	Manufacturer	Place of Origin
Vertical machining centre	VMC 850B	Shenyang Machine Tool Co., Ltd.	Shenyang, China
Quasi-continuous-wave fiber laser	RFL-QCW150/1500	Wuhan Raycus Fiber Laser Technologies Co., Ltd.	Wuhan, China
Six-axis robotic arm	CRP-RA18.20 J	Chengdu Canopus Robotics Co., Ltd.	Chengdu, China
Ultra-depth-of-field digital microscope	VHX-2000C	KEYENCE Corporation	Osaka, Japan
Ultrasound system	BT40 ultrasonic tool holder	Nanjing Puhang Machinery Development Co., Ltd.	Nanjing, China
Infrared thermal imaging camera	FLIR T630sc	FLIR Systems, Inc.	Wilsonville, OR, USA
Force measurement system	KISTLER 9257B	Kistler Instrumente AG/Kistler Group	Winterthur, Switzerland
Field-emission scanning electron microscope	JSM-IT800	JEOL Ltd.	Akishima, Tokyo, Japan
Laser Doppler vibrometer/non-contact vibrometer	KV-HE4525L	Nanjing KathMatic Technology Co., Ltd.	Nanjing, China
Laser spectral confocal microscope	KC-X1000	Nanjing KathMatic Technology Co., Ltd.	Nanjing, China

**Table 3:** Processing factors and levels used in the OFAT experiments.

Note	Laser Power *P* (W)	Scanning Speed *v*_s_ (mm/min)	Frequency *f* (kHz)	Amplitude *A* (μm)
I	300	200	30	3
II	400	300	34	5
III	500	400	38	7

**Table 4:** Complete experimental matrix for the OFAT experiments.

Test No.	Laser Power *P* (W)	Scanning Speed *v*_s_ (mm/min)	Frequency *f* (kHz)	Amplitude *A* (μm)	Investigated Factor
1	300	300	34	5	Laser power
2	400	300	34	5	Reference condition
3	500	300	34	5	Laser power
4	400	200	34	5	Scanning speed
5	400	400	34	5	Scanning speed
6	400	300	30	5	Frequency
7	400	300	38	5	Frequency
8	400	300	34	3	Amplitude
9	400	300	34	7	Amplitude

## Data Availability

The original contributions presented in this study are included in the article. Further inquiries can be directed to the corresponding authors.
